# Development of an arteriolar niche and self-renewal of breast cancer stem cells by lysophosphatidic acid/protein kinase D signaling

**DOI:** 10.1038/s42003-021-02308-6

**Published:** 2021-06-24

**Authors:** Yinan Jiang, Yichen Guo, Jinjin Hao, Rachael Guenter, Justin Lathia, Adam W. Beck, Reagan Hattaway, Douglas Hurst, Qiming Jane Wang, Yehe Liu, Qi Cao, Helen Krontiras, Herbert Chen, Roy Silverstein, Bin Ren

**Affiliations:** 1 Department of Surgery, University of Alabama at Birmingham School of Medicine, Birmingham, AL USA; 2 Biomedical Engineering, University of Alabama at Birmingham School of Medicine, Birmingham, AL USA; 3 Department of Cardiovascular and Metabolic Sciences, Cleveland Clinic, Cleveland, OH USA; 4 Department of Pathology, University of Alabama at Birmingham School of Medicine, Birmingham, AL USA; 5 Department of Pharmacology and Chemical Biology, University of Pittsburgh School of Medicine, Pittsburgh, PA USA; 6 Department of Biomedical Engineering, Case Western Reserve University, Cleveland, USA; 7 Department of Diagnostic Radiology and Nuclear Medicine, University of Maryland School of Medicine, Baltimore, MD USA; 8 O’Neal Comprehensive Cancer Center, University of Alabama at Birmingham, Birmingham, AL USA; 9 Department of Medicine, Medical College of Wisconsin, Milwaukee, Wisconsin, WI USA

**Keywords:** Breast cancer, Stem-cell research

## Abstract

Breast cancer stem cells (BCSCs) are essential for cancer growth, metastasis and recurrence. The regulatory mechanisms of BCSC interactions with the vascular niche within the tumor microenvironment (TME) and their self-renewal are currently under extensive investigation. We have demonstrated the existence of an arteriolar niche in the TME of human BC tissues. Intriguingly, BCSCs tend to be enriched within the arteriolar niche in human estrogen receptor positive (ER^+^) BC and bi-directionally interact with arteriolar endothelial cells (ECs). Mechanistically, this interaction is driven by the lysophosphatidic acid (LPA)/protein kinase D (PKD-1) signaling pathway, which promotes both arteriolar differentiation of ECs and self-renewal of CSCs likely via differential regulation of CD36 transcription. This study indicates that CSCs may enjoy blood perfusion to maintain their stemness features. Targeting the LPA/PKD-1 -CD36 signaling pathway may have therapeutic potential to curb tumor progression by disrupting the arteriolar niche and effectively eliminating CSCs.

## Abstract (web summary)

Jiang, Guo, Hao et al. investigate the role of the tumour microenvironment in breast cancer stem cell regulation and identify an arteriolar niche in which BCSCs tended to be enriched in human estrogen receptor positive breast cancer. Here, BCSCs are found to interact with arteriolar endothelial cells through LPA/PKD-1 signalling-mediated arteriolar differentiation of ECs and self-renewal of BCSCs.

## Introduction

Cancer stem cells (CSCs) are intimately connected with the vasculature and contribute to tumor growth, recurrence, and metastasis. In many advanced cancers, recurrence and metastasis are driven by a subpopulation of CSCs. These cells are characterized by their enhanced tumor-initiating and self-renewal capacities^1^. Al-Hajj and colleagues initially identified tumorigenic CSCs in breast cancer (BC) known as BCSCs^2^. The maintenance and expansion of BCSCs depend on both cell-intrinsic pathways and their interactions with the surrounding tumor microenvironment (TME). Tumors harbor CSCs in dedicated niches including vascular niches in BCs^3–7^. Niche stromal cells, such as tumor-associated endothelial cells (TECs), serve multiple functions within the TME^8^. Effective inhibition of angiogenesis within the TME, a hallmark of BC progression^9^, could disrupt the vascular niche and render BC dormant.

Aberrant tumor vascularity has repeatedly been shown to be important for satisfying voracious demands for nutrients by rapidly growing tumors, permitting metastasis, and serving as abnormal CSC niches^10,11^. A recent seminal study on glioblastoma demonstrates that more aggressive and chemo- and radio-resistant cancer cells enjoy the highest perfusion, which is inconsistent with the concept that tumor hypoxia is the major contributor for the aggressiveness^12^. Intriguingly, arterial networks may exist in solid tumors^13–15^ to provide oxygen and nutrients for tumor progression^12,14,16,17^. Previous studies have also shown that liver and lung metastases are arterialized^18,19^. In fact, angiogenesis was recently proposed as a vascular niche to provide such supportive cues as oxygen and glucose to maintain stem-like features via reciprocal signaling with CSCs^20^. However, much remains to be studied about how tumor vascular niches contribute to tumor progression and how heterogeneous perivascular niches participate in the regulation of CSCs. The signaling mechanisms that regulate the vascular niches and cancer stemness remain largely unknown.

Multiple signaling pathways are essential for angiogenesis and pro-arteriogenic reprogramming of ECs, including lysophosphatidic acid (LPA)/protein kinase D (PKD-1), which may regulate the vascular niche within the TME, in addition to promoting BC progression^21–25^. LPA as a lipid signaling mediator may promote arteriolar differentiation by the upregulation of such arteriogenesis-associated genes as ephrin B2 and DLL4^26^ via the PKD-1 pathway^27^. Recent studies also suggest that this pathway regulates the stem-like properties of cancer cells^28–30^. We hypothesized that the LPA/PKD-1 signaling axis regulates both arteriolar differentiation of the vascular endothelial cells and self-renewal of BCSCs.

In this study, we highlight the existence of the arteriolar niche within the TME of BCs. Intriguingly, CD44^+^/ALDH1^+^ BCSCs tended to be enriched within the arteriolar niche, a location where bi-directional interactions occurred between arteriolar ECs and BCSCs. Furthermore, LPA/PKD-1 signaling induced arteriolar differentiation that may contribute to arteriolar niche development and directly promoted self-renewal of BCSCs. These studies indicate that the LPA/PKD-1 signaling may play an essential role in tumor progression by nurturing the development of an arteriolar niche to enhance CSC self-renewal and directly promoting stemness features of cancer cells.

## Results

### Enrichment of breast cancer stem-like cells in the arteriolar niche

To determine the distribution of BCSCs in the TME, we interrogated both the presence and localization of these cells in BC tissues from patients using immunohistochemistry. BC cells that demonstrate a marker profile of CD44^+^/CD24^−^/ALDH1A1^+^ are commonly accepted as BCSCs^2^. We detected CD44^+^/CD24^−^/ALDH1A1^+^ BCSCs within the TME (Supplementary Figure 1).

**Fig. 1:**
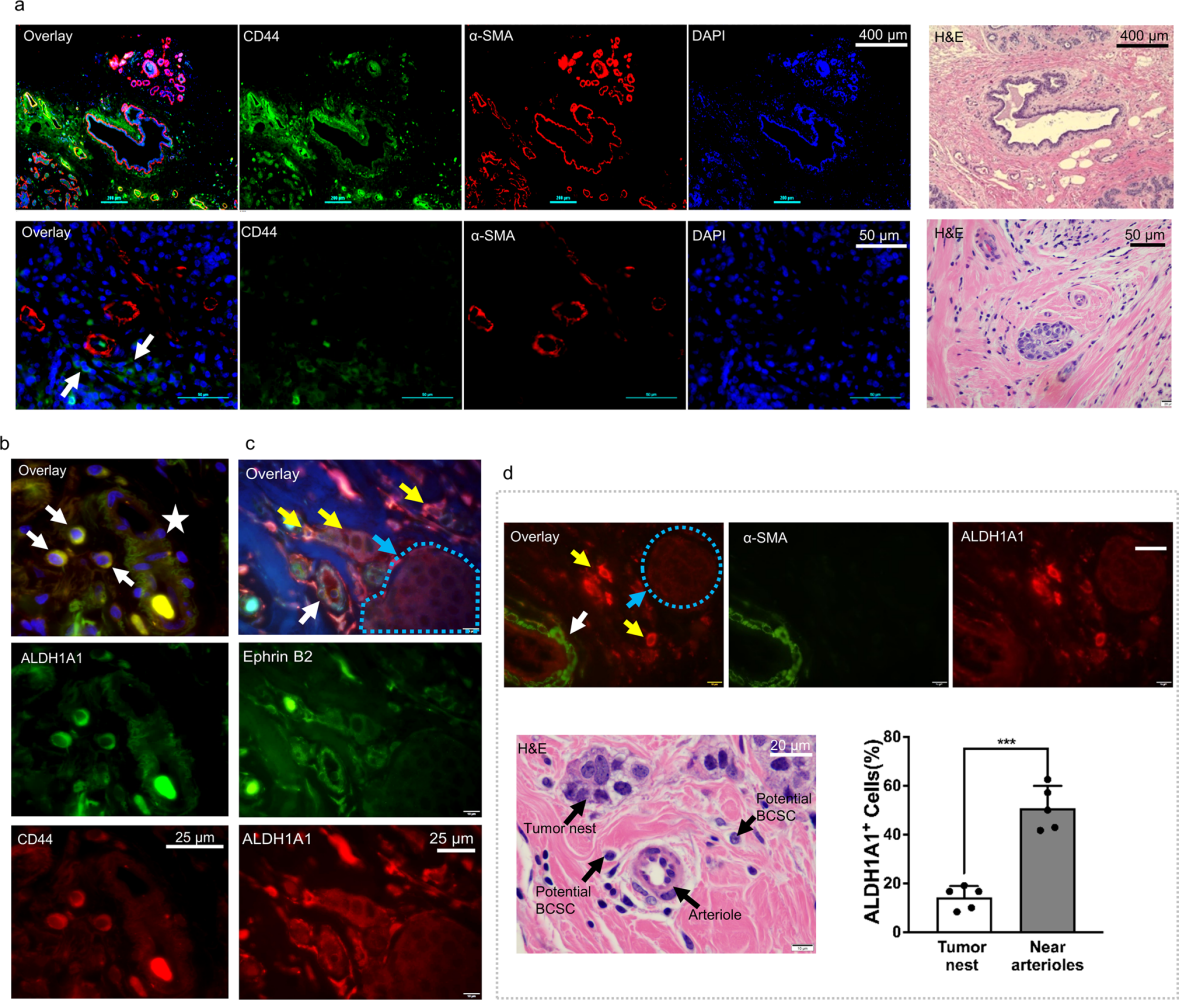
Breast cancer stem-like cells are highly enriched within the arteriolar niche. **a** CD44^**+**^ cancer cells within a vascular niche (white arrows), as shown by staining with α-SMA (red) in human ER^**+**^ breast cancer. DAPI (blue) is used for nuclei staining. Normal tissue control (upper panel) refers to the tissues adjacent to breast cancer. Tissue structures are shown by H&E staining. Scale bar = 400 µm (upper panel) or 50 µm (lower panel). **b** BCSCs in a human BC specimen within TME. Human ER + BC specimen was co-stained with anti-CD44 and anti-ALDH1A1 antibodies, followed with appropriate second antibodies, and mounted with DAPI. Images were acquired using an Eclipse T*i*2^®^Nikon microscope and overlaid using NIS Elements software. BCSCs are indicated by white arrows and the blood vessel-like structure is shown by a white star. No CD24 was detected. Shown are representative images. Bar = 25 µm. **c** Ephrin B2 and ALDH1A1 co-staining in human ER^**+**^BC specimens. Ephrin B2 was stained green and ALDH1A1 red. White arrows show ephrin B2 positive arteriole, yellow arrows indicate ALDH1A1^+^ cancer cells, and tumor nest is indicated by the blue dashed circle. Cells in the cancer nests as indicated did not show significant ALDH1A1 staining (blue arrow). Scale Bar = 10 µm. **d** Quantification of ALDH1A1^+^ BC cells within the arteriolar niche. The arteriole is indicated by the white arrow, ALDH1A1^+^ BCSCs were shown by yellow arrows, and tumor nest was indicated by the blue dashed circle and BCs with low ALDH1A1 expression occurred within the tumor nest (blue arrow). H&E staining as a tissue structure control. Scale bar = 10 µm. BC cells were randomly counted under an Olympus BX60 fluorescence microscope linked with a CCD camera. The standard for examining cell locations was: (1) closed to the artery is defined as ALDH1A1^+^ BC cells within 50 µm from the SMA^+^ small arteries, and (2) close to the cancer nest refers to ALDH1A1^+^ BC cells locating cancer cell nest more than 50 µm from the SMA^+^ small artery. Up to 30 cancer cells were randomly counted within 50 µm × 50 µm area with five repetitions. The yellow arrows indicate ALDH1A1^+^ BCSCs. The dotted blue circle indicates a tumor nest containing BC cells to determine BCSCs that were localized more than 50 µm from the α-SMA^+^ arterioles. The bar graph shows relative numbers of ALDH1A1^+^ BCSCs that were related to either arteriolar niche or tumor nests in the BC tissues as detected by immunofluorescence microscopy. ***P < 0.001 compared with cells in the tumor nest.

To more precisely define the location within the arteriolar niche where BCSCs tend to be enriched, we stained BC tissues from patients for the presence of ALDH1A1, ephrin B2 (an arterial EC marker essential for arterial function), and α-smooth muscle actin (α-SMA) for smooth muscle cells in arterioles (Fig. 1a–d). ALDH1A1 served as a marker for BCSCs, whereas the co-staining of ephrin B2 and α-SMA served as markers of the arteriolar niche. Intriguingly, ALDH1A1^+^-BCSCs tended to be localized to the arteriolar niche rather than in the tumor nest (Fig. 1d). Additionally, in a preclinical setting, syngeneic mice serving as models of BC were treated with LPA and were found to have expanded arteriolar networks as demonstrated by a significant increase in α-SMA^+^ blood vessels within the TME (Supplementary Figure 2). Our current data thus suggest that BCSCs tend to be enriched within the arteriolar niche and that these cells may be plastic as they expressed high levels of ALDH1 marker^31,32^, thereby contributing to tumor progression.

### Crosstalk between arteriolar ECs and BCSCs

**Fig. 2:**
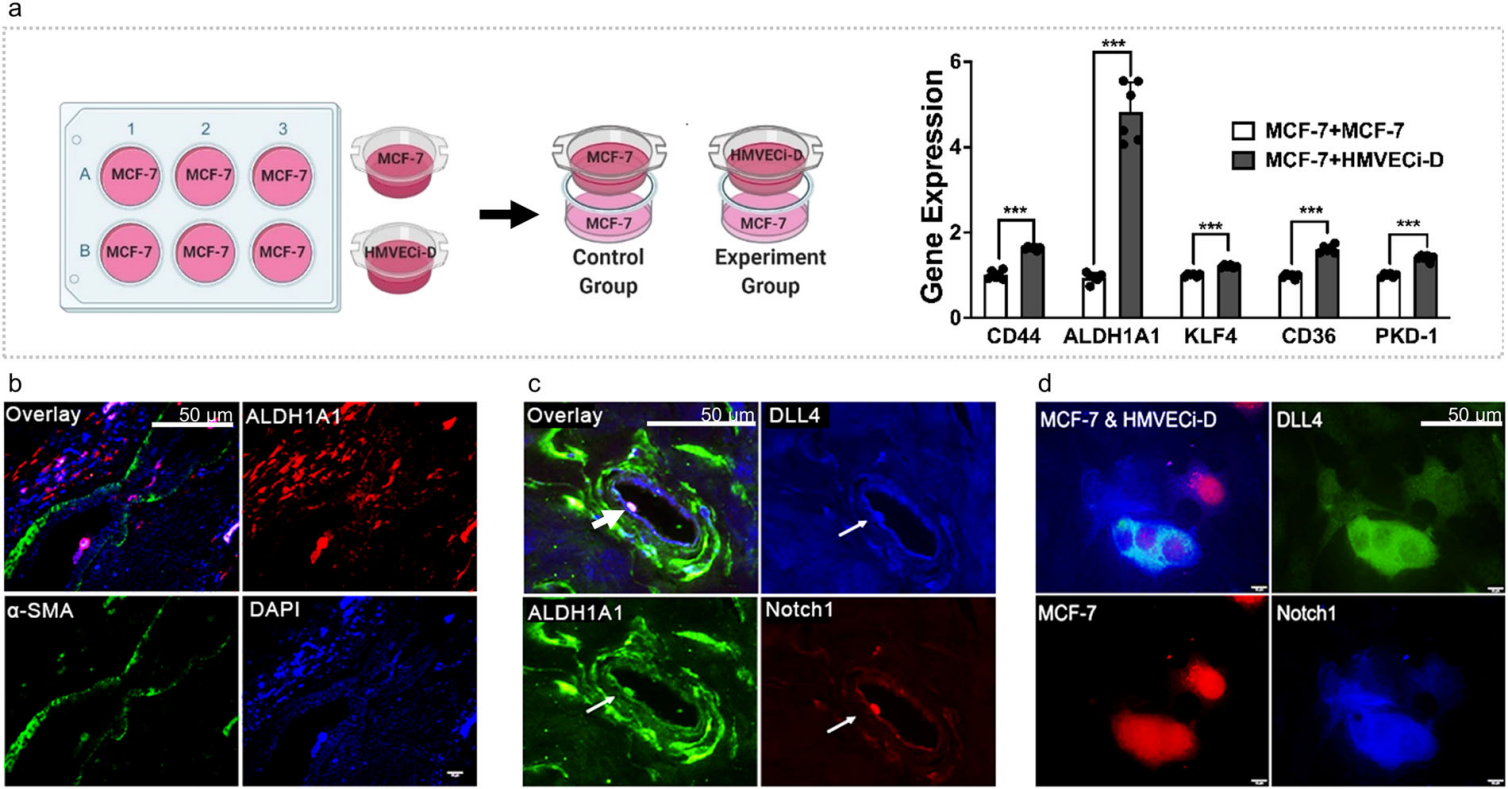
Indirect and direct interactions of breast cancer stem-like cells with the arteriolar endothelium within TME. **a** Coculture of breast cancer cells and endothelial cells increased the expression of BCSC markers and stemness-associated genes. Total RNA in MCF-7 cells cultured in a six-well plate was collected for detection of stemness-associated genes at mRNA levels by RT-qPCR. The results of triplicate experiments are shown as mean ± SEM. ^***^P < 0.001, ^**^P < 0.01, or ^*^P < 0.05 compared with the control. **b** An ALDH1A1^+^ BCSC was attached to the arteriolar endothelium within the vascular lumen. The human BC specimen was stained with ALDH1A1 and alpha-smooth muscle actin (α-SMA) antibodies used, followed by appropriate secondary antibodies and mounted with DAPI. Scale bar = 50 µm. **c** An ALDH1A1^+^ cell that expresses high levels of Notch1 and DLL4 interacts with DLL4^+^ endothelium in the arteriolar vessel. Human breast cancer specimens were processed and stained with Notch1, DLL4, and ALDH1A1 antibodies followed by appropriate secondary antibodies. Scale bar = 50 µm. **d** MCF-7 cells transduced with DsRed interacted with an HMVEC. MCF-7 cells transduced with Ds-Red that were grown in complete MammoCult^™^ Media for 5 days were cocultured with HMVECi-Ds that expressed with DLL4 at the ratio of 2:1 in DMEM for 48 h. The cells were stained with Notch1 and DLL4 antibodies, followed by appropriate secondary fluorescence antibodies. Scale bar = 50 µm. The images were taken with an Olympus BX60 fluorescence microscope, and representative images are shown.

To further investigate the relationship between BC cells and the arteriolar niche, we sought to determine whether BC cells interact with surrounding arteriolar ECs indirectly, directly, or both. As a test for indirect crosstalk, we used transwell chambers to coculture estrogen receptor-positive (ER^+^) BC cells (MCF-7) and human microvascular ECs (HMVECs) that express delta-like ligand 4 (DLL-4), a marker of well-differentiated arteriolar ECs and a ligand involved with Notch1 signaling. ER^+^ BC cells grown in coculture with DLL4^+^-HMVECs had higher levels of gene expression of cancer stemness-related genes (*CD44*, *ALDH1A1*, *KLF4*, and *CD36*) and *PKD-1*, as compared to ER^+^ BC cells grown in monoculture (Fig. 2a). These results suggest that BCSCs may take advantage of the arteriolar niche for their maintenance and self-renewal.

In addition, PKD-1 signaling may play a dual role in both endothelial cells and BC cells to promote BCSC expansion (Fig. 4d, e). To test for direct crosstalk, we three-dimensionally cocultured (Supplementary Fig. 3a) GFP-labeled BC cells (E0771) (Supplementary Fig. 3b) with Ds-Red-labeled HMVECs (Supplementary Fig. 3c) and observed the interactions of MVEC and BC cells in the margin area (Supplementary Fig. 3a).

Notch1, an important molecule in human BCs, regulates SC self-renewal^33,34^, and EC Notch1 promotes metastasis^35^ in addition to its role in arteriolar differentiation^36,37^. We next characterized BCSCs present in the tumor vasculature in vivo. Using human ER^+^ BC specimens analyzed by immunofluorescence microscopy, we found the existence of Notch1^+^ BC cells within the microvasculature, suggesting a direct interaction with arteriolar ECs, which was supported by a vascular-like structure consisting of DLL4^+^ and DLL4^+^/Notch1^+^ cells and blood vessel-like structure composed of DLL-4^+^ cells (Supplementary Fig. 4). Moreover, an ALDH1A1^+^ BC cell directly attached to the arteriolar endothelium (Fig. 2b).

DLL-4, the ligand to the Notch1 receptor, has been shown to be upregulated in the tumor vasculature^38^. This ligand is also expressed by arteriolar ECs in the vascular system^36,39^. To determine whether the Notch1 pathway is involved in mediating the crosstalk between arteriolar endothelium and BCSCs, we analyzed human BC tissue from patients for the expression of ALDH1A1, Notch1, and DLL-4. Interestingly, ALDH1A1^+^ BCSCs that co-expressed Notch1 showed direct contact with the tumor endothelium that expressed DLL4 in a tumor arteriole (Fig. 2c). This interaction indicates direct crosstalk between arteriolar ECs and BCSCs. To further confirm this interaction, we co-cultured enriched-BCSCs with MVECs that showed high levels of DLL-4 expression. The results demonstrated that Notch1^+^ BC cells were likely to directly interact with the DLL-4^+^ MVECs (Fig. 2d). These data suggest that arteriolar differentiation of vascular ECs may bridge the crosstalk between BCSCs and ECs via Notch signaling.

### LPA/PKD-1 signaling in arteriolar differentiation of vascular endothelial cells

Arteriogenic gene regulation and arterial differentiation are regulated by a MAP kinase/Erk signaling^40,41^, while Erk-related PKD-1 is integral to angiogenesis through its interactions with the VEGF and CD36 signaling pathway^21,25,42–45^. PKD-1 not only activates Erk^46^ but also transduces PLCγ1 signaling^47^, which is crucial in arterial differentiation^48^.

**Fig. 3:**
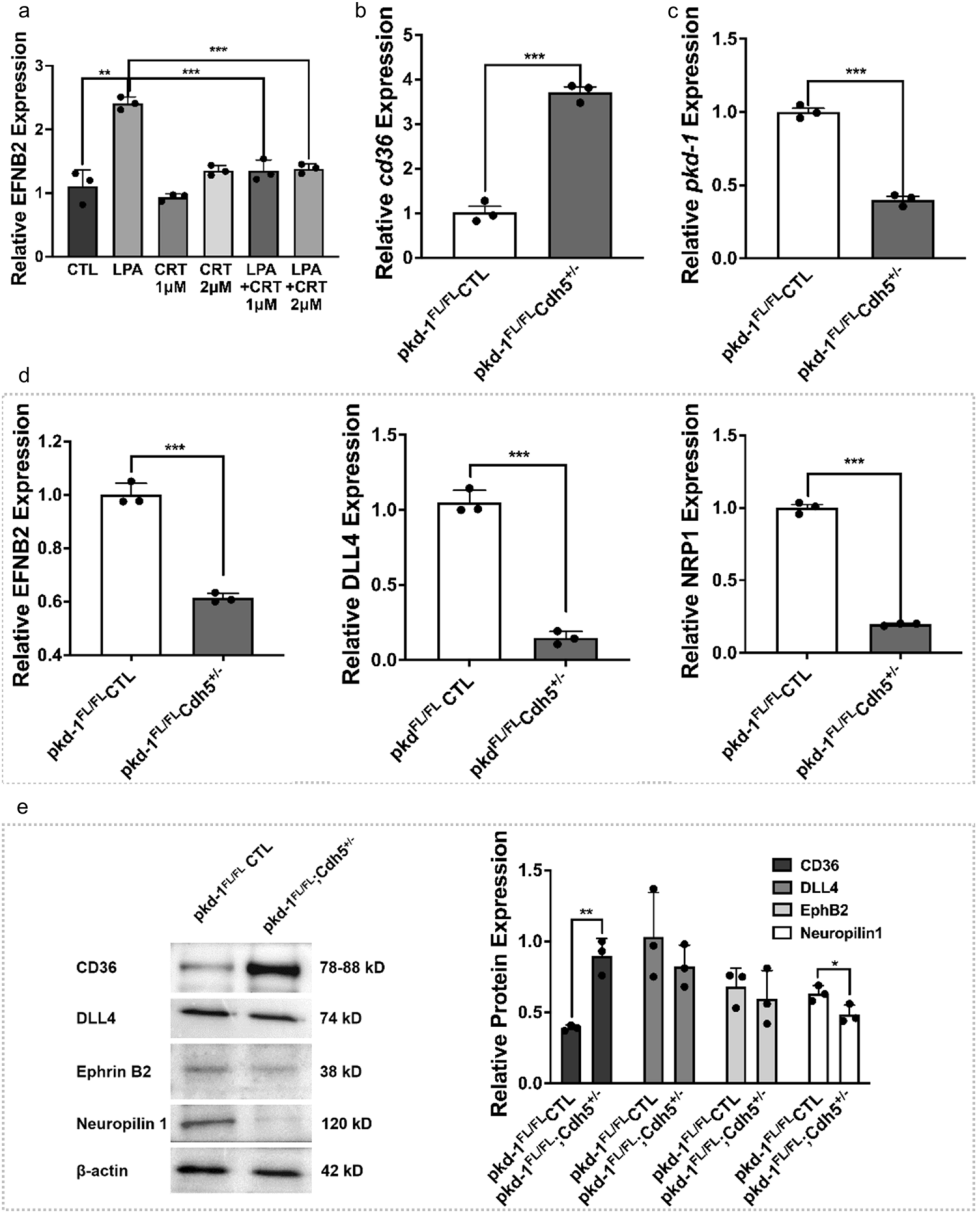
Regulation of CD36 and arteriogenic gene expression via PKD-1 signaling in vascular endothelial cells. **a** HMVECi-D cells were cultured in an endothelial medium (DMEM medium with 5% MVGS and 5% FBS). After starvation in serum-free DMEM medium for 6–8 h, the cells were treated with 10 µM of LPA, and/or 1 or 2 µM of CRT0066101 in serum-free DMEM medium for 24 h under 5% CO_2_ and 37 °C. The total RNA was extracted and the mRNA levels of arterial gene ephrin B2 were detected by RT-qPCR. **b** Primary lung endothelial cells (ECs) were isolated from the control and EC-specific *pkd-1* knockout mice. Passage 1 ECs were used for extraction and purification of total RNA. mRNA levels of *pkd-1* were detected by RT-qPCR and relative expression was compared between the control and *pkd-1*-deficient ECs. **c** Increased expression of *cd36* in *pkd-1*-deficient lung ECs. Lung ECs were isolated from the control and EC-specific *pkd-1* knockout mice. Passage 1 lung ECs were used for extraction and purification of total RNA. mRNA levels were detected by qPCR and relative expression was compared between ECs from the control and EC-specific *pkd-1* knockout mice. **d** Decreased expression of arteriogenic genes in *pkd-1*-deficient lung ECs. Primary lung ECs were isolated from the control and EC-specific *pkd-1* knockout mice. Passage 1 ECs were used for extraction and purification of total RNA. mRNA levels were detected by RT-qPCR and relative expression was compared between the control and *pkd-1*-deficient ECs. **e** The cell lysate was extracted from passage 1 primary lung ECs that were isolated from EC-specific PKD-1 and control mice for detection of protein expression by Western blotting. Triplicate experiments were performed and levels of protein expression assessed by densitometry with Image J. ^*^P < 0.05, ^**^P < 0.01, or ^***^P < 0.001 vs. control.

To determine whether LPA/PKD-1 signaling is essential for arteriolar differentiation of vascular ECs, we treated HMVECi-D cells with either LPA and/or a PKD inhibitor and then examined the expression of arterial genes by RT-qPCR. Exposure to LPA significantly increased the gene expression of ephrin B2 relative to the control, an effect that was reversible upon treatment by a PKD inhibitor (Fig. 3a). The lipid signaling mediator LPA activates PKD-1 to regulate angiogenesis by suppressing transcription of CD36, a well-established angiogenic regulator that initiates antiangiogenic responses^49^ and mediates ischemic injury^50^. To precisely define the role of PKD-1 signaling in the regulation of arterial gene expression, we isolated primary lung ECs from both wild-type control (Flox) mice and the endothelial-specific-*pkd-1* knockout mice. We confirmed that the gene expression of PKD-1 was significantly downregulated in PKD-1-deficient primary lung ECs (Fig. 3b) whereas the expression level of CD36, the angiogenesis inhibitor in ECs that may participate in arteriolar differentiation^21^, was significantly increased (Fig. 3c). Correspondingly, the expression of such arteriogenic gene signature including ephrin B2, DLL-4, and neuropilin 1 was significantly decreased compared to controls (Fig. 3d). Furthermore, there was a significant decrease in the expression of neuropilin 1, while CD36 expression was significantly increased at the translational level when the *pkd-1* gene was deleted in lung ECs (Fig. 3e). Additionally, the protein expression of DLL-4 and ephrin B2 tended to be decreased though it did not reach statistical significance (Fig. 3e). These studies suggest that LPA/PKD-1 signaling is critical for the arteriolar differentiation of vascular ECs.

### LPA/PKD-1 signaling in self-renewal of BCSCs

In cancer compartments, LPA signaling may protect mesenchymal SCs from apoptosis^51^ and promote the expansion of CSCs^28^ as well as contribute to BC progression^22,52,53^. To test the hypothesis that LPA signaling promotes the expansion of BCSCs via PKD-1, we examined the expression of PKD-1 in a syngeneic BC model in mice with high-fat diet (HFD)-induced obesity that generates excessive LPA^22^ and in mice on a normal diet with or without the administration of LPA. Immunohistochemical assays demonstrated that HFD increased PKD-1 levels in the BC tissues. However, LPA treatment elevated PKD-1 levels in the BC tissues to an even greater extent and increased the percentage of the PKD-1^+^ megakaryocyte (MC) and/or polykaryocyte (PC) (Supplementary Fig. 5). To further determine the expression level of PKD-1 in BC tissues of human patients, we examined pathological specimens resected from patients with ER^+^ BC. Intriguingly, PKD-1^+^ cancer cells presented a distinct distribution between different patients. A subpopulation of BC cells showed high levels of PKD-1 expression, particularly in the tumor nests (Supplementary Fig. 6a), whereas singular BC cells had a moderate expression of PKD-1 (Supplementary Figs. 6a–d and 7).

To validate the association of PKD-1 signaling with BC stemness in vivo, we utilized immunofluorescence microscopy to observe co-expression of CSC markers and PKD-1. As expected, we first identified BC cells that were positive for both PKD-1 and CD44 in a syngeneic mouse BC model (Supplementary Fig. 6b). The results from animal studies were also supported by those in patients’ BC specimens (Supplementary Fig. 6c). Interestingly, the individual BC cells with positive co-staining for both PKD-1 and CD44 were located outside of the tumor nest in patient BC specimens (Supplementary Fig. 6c), suggesting that PKD-1 signaling may promote metastatic potential via maintenance of CSC phenotype.

CD36 is a driver for CSC phenotype and expression of CD36 in CSCs increases metastatic potential^54,55^. Intriguingly, we also observed that a few CD36^+^ cells existed in ALDH1A1^+^- and CD44^+^-mouse BC cells that were transduced with GFP (Supplementary Fig. 6e). While in mouse BC tissues some BC cells also co-stained positively for both PKD-1 and CD36 (Supplementary Fig. 6F). Furthermore, the Oncomine database analysis^56^ demonstrated that the expression of CD36, a driver for tumor progression and metastasis^22,54,55^ was associated with tumorigenesis in an aggressive human ER^+^ BC cell line (Supplementary Fig. 8a). In this study, we observed that CD36 levels were increased in individual cells or tumor nests in ER^+^ BC patient specimens (Supplementary Fig. 6d and Supplementary Fig. 8b).

**Fig. 4:**
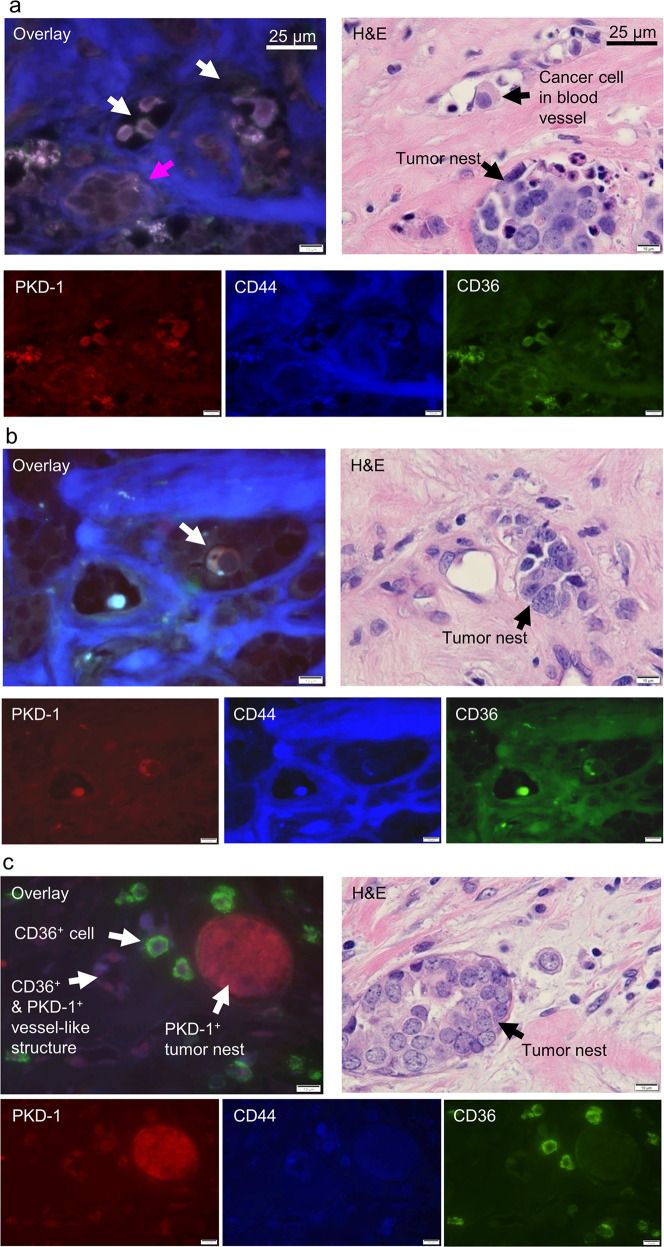
Distribution of breast cancer stem cells expressing both PKD-1 and CD36. **a** Immunofluorescence microscopy showed that PKD-1^+^ or CD36^+^ cancer stem-like cells (CD44^+^ BCSCs) were located within the blood vessel (white arrows) or tumor nest (pink arrow). H&E staining as a control for tissue structure, and a tumor cell existed within the blood vessel and tumor nest exists in the tumor microenvironment (black arrows). Scale bar = 25 µm or 10 µm. **b** Immunofluorescence microscopy showed that cancer stem-like cells with positive PKD-1 or CD36 expression were located nearby the tumor blood vessels (white arrow). H&E staining showed tissue structure of the breast cancer and tumor nest was indicated by a black arrow. Scale bar = 25 or 10 µm. **c** A small subset of CSCs expressing moderate levels of PKD-1 but high levels of CD36 were located outside of cancer nest that also expressed high levels of PKD-1 but moderate levels of CD44 in human BC. A small subset of CSCs expressed moderate levels of PKD-1 and CD44 but minimal levels of CD36 and appeared to form a vessel-like structures. A tumor nest showed strong PKD-1 expression (white arrows). H&E staining as a control for tissue structure. Scale bar = 25 or 10 µm.

To further determine the association of PKD-1 with BC stemness, we stained the human BC tissues with PKD-1, CD36, and CD44. We found that a subset of CD44^+^ BCSCs with high expression levels of PKD-1 and CD36 were localized within or near blood vessels and tumor nests (Fig. 4a, b). BCSCs with high CD36 and moderate PKD-1 expression were localized outside of the tumor nest and had invaded into the surrounding tissue (Fig. 4c), whereas the BCSCs with low CD36 and moderate PKD-1 expression appeared to form a vessel-like structure or capillary (Fig. 4c). The data suggest that PKD-1 signaling may promote the CSC phenotype via CD36, which may be associated with the formation of an arteriolar niche within the TME.

**Fig. 5:**
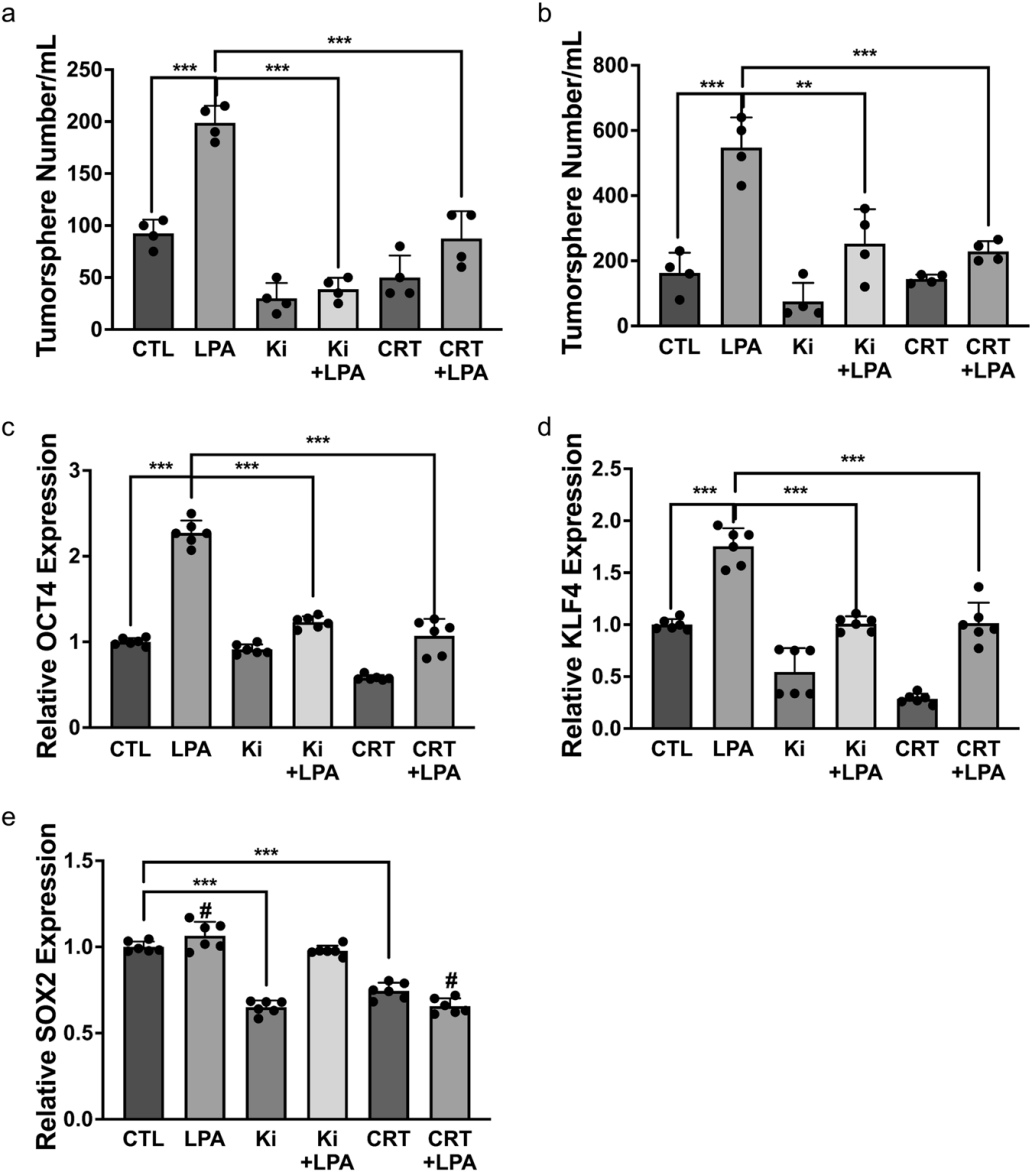
LPA-PKD-1 signaling in breast cancer stemness. **a** Tumorsphere formation of mouse BC cells. CD44^+^ E0771 cells were cultured in complete MammoCult™ Medium with the treatment of 10 µm LPA, 1 µm CRT0066101 (PKD inhibitor), 1 µm Ki16425 or their combination for 7 days. **b** Tumor sphere formation of ER^+^BC cells. Human ER^+^BC cells (MCF-7) were cultured in complete MammoCult^™^ Medium with the treatment of 10 µm LPA, 1 µm CRT0066101 (PKD inhibitor), 1 µm Ki16425 or their combination for 7 days. The mammary spheres were counted under the OLYMPUS CK30 microscope, triplicate experiments were performed, and the results are shown as the mean value $\pm$ SEM. ^***^P < 0.001 compared with control or LPA treatment. **c** LPA-PKD-1 signaling stimulated the expression of Oct4 in ER^+^BC cells. ^**^P < 0.01, compared with control or LPA treatment. **d** LPA-PKD-1 signaling stimulated the expression of KLF4 in ER^+^BC cells^.^ ***P < 0.001, compared with control or LPA treatment. **e** Sox2 expression was regulated differently by LPA/PKD-1 signaling. Total RNA was extracted from MCF-7 tumorspheres with different treatments, and mRNA levels were assayed with RT-qPCR. The results of triplicate experiments are shown as mean ± SEM. ^***^P < 0.001 or ^#^*P* < 0.001 compared with the control or LPA treatment.

LPA is known to function via activation of the PKD-1 pathway in both ECs and cancer^21,22,25,57^. In addition, studies suggest that LPA/PKD-1 signaling may be associated with the stemness of cancer cells^28–30^. We sought to assess whether LPA/PKD-1 signaling is critical for the maintenance and self-renewal of BCSCs by using mammosphere formation assays that can identify functional BCSCs in vitro. By taking advantage of the anchorage-independent property of BCSCs for cell expansion and enrichment in ultra-low attachment plates, we initially optimized the growth conditions to observe the role of LPA/PKD-1 signaling in BCSCs grown as mammospheres. After optimizing growth conditions, we reproducibly showed that LPA/PKD-1 signaling significantly promoted the self-renewal of BCSCs in both mouse (Fig. 5a) and human (Fig. 5b) BC cells. To define the association between LPA/PKD-1 signaling and BCSCs, we analyzed changes in the expression of stemness-associated genes in mammospheres treated with either LPA or PKD inhibitor (CRT). BCSCs exposed to LPA significantly increased the expression of *Oct4* (Fig. 5c) and *KLF4* (Fig. 5d), genes that sustain self-renewal capacity^58,59^. Moreover, the addition of either an LPA antagonist or a PKD inhibitor attenuated the LPA-induced expression of *Oct4* (Fig. 5c), and *KLF4* (Fig. 5d).

Unexpectedly, LPA treatment did not increase the expression of *Sox2*, a transcription factor that is expressed in CSCs and mediates resistance toward established cancer therapies^60^ (Fig. 5e). However, inhibition of endogenous LPA/PKD-1 signaling by either LPA antagonism or PKD inhibition decreased the gene expression of Sox2. Furthermore, LPA exposure recovered LPA antagonist-mediated, but not PKD inhibitor-mediated, Sox2 downregulation (Fig. 5e), suggesting other pathways rather than PKD-1 signaling may regulate LPA-modulated Sox2 expression.

**Fig. 6:**
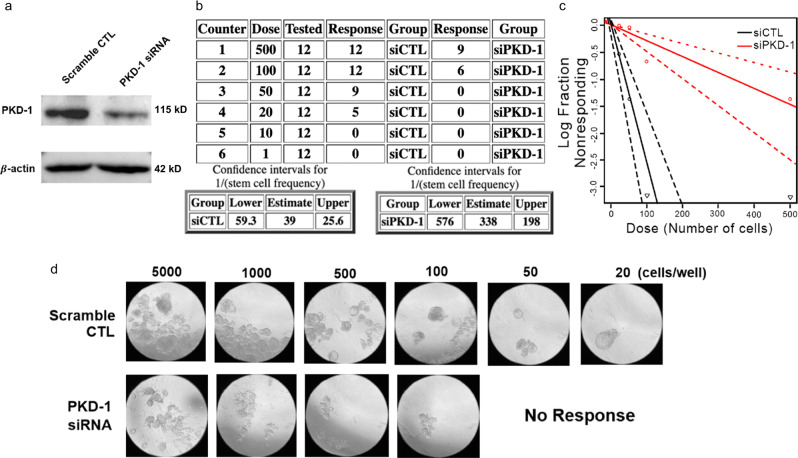
PKD-1 is required for tumorigenicity by tumorsphere formation efficiency assay. **a** Immunoblotting assay indicates that the PKD-1 expression decreased when transfecting MCF-7 with PKD-1 siRNA as compared with the scramble control (CTL). **b** MCF-7 with PKD-1 depletion significantly reduced tumorsphere formation efficiency. **c** Quantitation graph shows that the number of tumorspheres formed in each well with the seeding density starting from 500 to 20 cells/well by statistical analysis. **d** Representative images for tumorsphere formation in MCF-7 cells subjected to siRNA scramble control (CTL) and PKD-1 siRNA and with different seeding densities.

To validate the role of LPA/PKD-1 signaling in supporting BCSC stemness, we evaluated the impact of PKD-1 depletion on BCSC tumor-initiating potential in well-established in vitro limiting dilution tumor assays. Transfection of siRNA demonstrated an efficient knockdown of endogenous PKD-1 expression at the protein level (Fig. 6a). Furthermore, knocking down PKD-1 expression significantly impaired the frequency of repopulation of the cells when compared with the control (Fig. 6b–d). These results suggest that LPA/PKD-1 signaling may be critical in BCSC maintenance and expansion as well as in tumor initiation capacity.

**Fig. 7:**
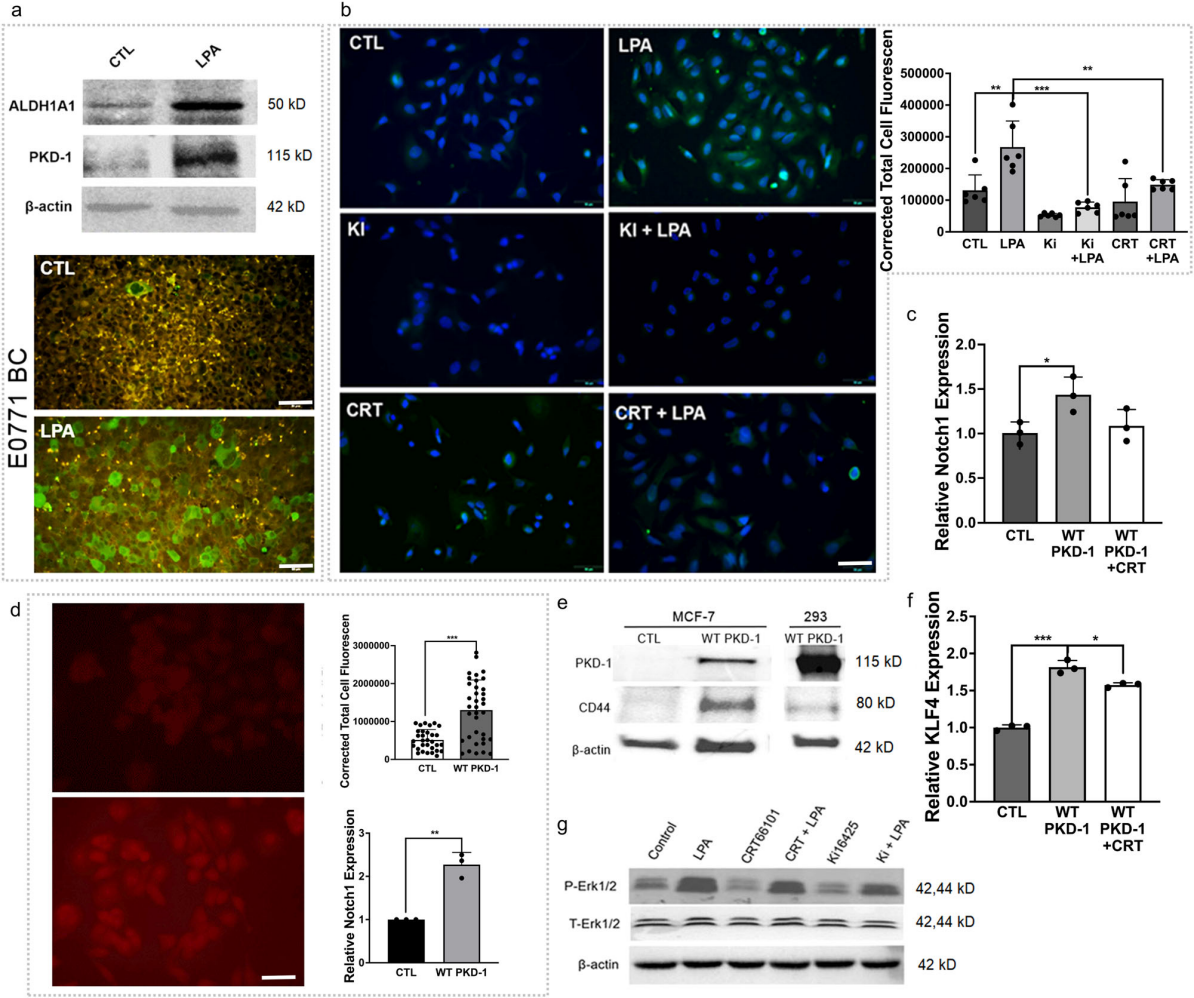
LPA-PKD-1 signaling pathway in self-renewal and plasticity of breast cancer stem cells. **a** Induction of ALDH1A1 expression in BC cells (upper panel). The E0771 cell was cultured in RPMI-1640 medium with 10% FBS and 1% penicillin/streptomycin. The following starvation in serum-free medium for 6 h, the cells were treated with LPA (10 μM) for 24 h, the cell lysates were collected for Western Blot. Duplicated experiments were performed, and shown is a representative result; ALDH1A1 expression in implanted E0771 BC (lower panel). Immunofluorescence microscopy was used to detect ALDH1A1^+^BC cells in tumor tissues. Representative images are shown. Scale bar = 50 µm. **b** MCF-7 cells were exposed to 10 µm LPA, 1 µm CRT0066101 (PKD inhibitor), 1 µm Ki16425 (LPA_1,3_ antagonist), and their combinations for 24 h. The control and treated MCF-7 cells were incubated with ALDH1A1 antibody followed by an appropriate secondary antibody. Representative images are shown from triplicate experiments. Fluorescence intensity was measured by ImageJ and calculated by the corrected total cell fluorescence (CTCF). The relative expression was shown as the mean ± SEM. ^**^P < 0.01 compared with the control. Scale bar = 50 µm. **c** Total RNA was extracted from the MCF-7 control, those transduced with wild-type PKD-1 (PKD-WT) or the PKD-1-transduced MCF-7 cells that were treated with PKD inhibitor CRT0066101. The mRNA levels were assayed by RT-qPCR. ^*^P < 0.05 compared with the control. **d** MCF-7 cells transduced with wild-type PKD-1 (PKD-WT) were stained with Notch 1 antibody, followed by an appropriate secondary antibody. The fluorescence intensity was measured by ImageJ and calculated by the CTCF. Representative images are shown. Scale bar = 50 µm. In addition, the total RNA was collected from the control and MCF-7 with overexpressing PKD-WT were collected and Notch1 mRNA levels were assayed with RT-qPCR. Triplicate experiments were performed and results shown as the mean ± SEM. ^**^P < 0.01 or ^***^P < 0.001 compared with the control. **e** Overexpression of PKD-WT increased protein **e**xpression of CD44 in BC cells. MCF7 cells were transduced with PKD-WT and protein lysate was collected for Western blots. MCF7 without PKD-WT or 293 T cells transduced with PKD-WT as an experimental control. **f** Overexpression of PKD-WT induced KLF4 expression in BC cells. MCF7 cells transduced with PKD-WT were starved in serum-free DMEM media overnight and treated with/without CRT0066101 (0.5 µm) for 24 h. Total RNA was extracted for the detection of KLF4 mRNA levels by RT-qPCR. *P < 0.05 or ***P < 0.001. **g**. MCF7 cells were serum-free starved overnight and then treated with LPA (10 µm) and/or LPA antagonist Ki16425 (1 µm) or CRT0066101 (1 µm) for 24 h. The cell lysates were collected for protein expression by Western Blot. Duplicate experiments were performed.

LPA treatment of mouse BC cells and BC-bearing mice suggested that PKD-1 signaling likely increased the expression of ALDH1A1 (Fig. 7a). To further define the role of the LPA/PKD-1 signaling pathway in BC progression via ALDH1, we exposed human BC cells to either LPA, an LPA antagonist, or a PKD inhibitor and examined changes in ALDH1A1 expression. Immunofluorescence microscopy further showed that the expression of ALDH1A1 was significantly increased in response to LPA/PKD-1 signaling (Fig. 7b).

Given that Notch1 activity plays a pivotal role in the stemness and progression of ER^+^ BC^61–63^ and ECs can interact with BC via the Notch1 pathway^7,63^, we tested whether PKD-1 signaling can impact Notch1 expression by transducing BC cells with PKD-1. Overexpression of PKD-1 significantly increased the expression of Notch1 at both mRNA and protein levels in BC cells (Fig. 7c & d), along with an increased expression in CD44 (Fig. 7e) and KLF4 (Fig. 7f). To provide a mechanistic explanation for LPA-induced BC stemness, we examined the downstream of PKD-1 signaling MAPK/Erk1/2. The results showed that LPA-mediated MAPK/Erk1/2 phosphorylation was inhibited by a PKD inhibitor (Fig. 7g), suggesting that LPA/PKD-1 signaling may stimulate BC stemness via MAPK/Erk1/2-mediated stemness-associated gene transcription.

## Discussion

Current anti-angiogenic therapies are ineffective under experimental conditions^64,65^ and show limited efficacy in the clinic^16,66^ despite the resulting stabilization of disease and increased progression-free survival. Many factors contribute to the resistance of antiangiogenic therapy^67^. The Dvorak group proposed that the resistance may result from established mature vasculature^17^. Therefore, the heterogeneity of vascular niches in the TME should be considered in order to achieve better antiangiogenic efficacy^14,16^. Among potential approaches urgently needed may be the targeting of different vascular components, particularly late-forming blood vessels such as feeding arteries and de novo arteriogenesis^14,17^, which may function as a unique vascular niche for CSCs.

CSCs can accumulate in the perivascular regions within the TME^23^. However, vascular niche preferences are not well-understood. The interactions between CSCs and their heterogeneous vasculature^14,15^ may determine CSC development and fate. In addition, circulating tumor cells contain a distinct subset of CSCs that bear metastasis-initiating capabilities^31^ whereas CSCs prefer to exist in arterial blood to venous blood^68^, and arterialization may contribute to liver and lung metastases^18,19^. These studies strongly suggest that the arteriolar niche contributes to the maintenance and expansion of CSCs. Consistent with these observations, our study highlights the existence of the arteriolar niche in the TME of human ER^+^BCs. Intriguingly, aggressive ALDH1^+^-BCSCs tend to be enriched in the arteriolar niche. These BCSCs may enjoy a high level of perfusion as shown by Kumar et al.^12^. This is also supported by the fact that an aggressive BCSC population can be developed in vitro through cyclic-reoxygenation^69^. Moreover, arteriolar ECs may crosstalk with BCSCs via the Notch signaling pathway, directly and indirectly, in which LPA/PKD-1 signaling-mediated arteriolar differentiation may play an important role. Our study thus uncovers a previously underappreciated vascular type within the TME that promotes tumor progression preferentially by increasing the perfusion of nutrients and oxygen to the surrounding CSCs.

**Fig. 8:**
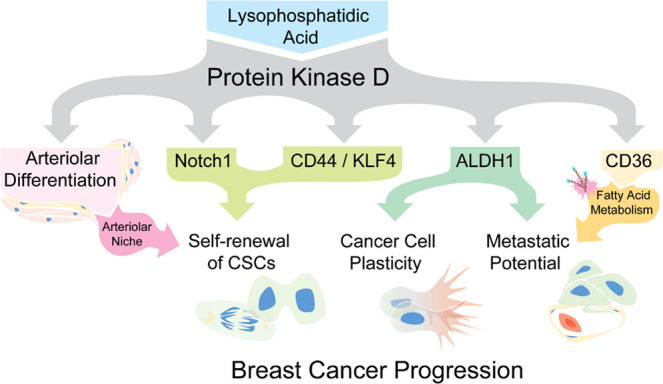
LPA/PKD-1 signaling in the development of an arteriolar niche within the tumor microenvironment and cancer stem cell expansion in estrogen receptor-positive breast cancer. A proposed mechanistic model shows that the LPA/PKD-1 signaling regulates arteriolar differentiation within TME, CSC plasticity, and BC progression.

Furthermore, the crosstalk between BCSCs and arteriolar ECs via Notch signaling may contribute to changes in CSC phenotypes^7,63,70^, in which PKD-1 signaling-mediated arteriolar differentiation may be indispensable by the development of an arteriolar niche. Although distinct vascular niches are likely needed to regulate BCSCs, we found that LPA/PKD-1 signaling-mediated DLL4 expression in the arteriolar ECs facilitates direct EC interaction with Notch1^+^ stem-like cells. The interaction of Notch-DLL4 may be an important mechanism by which the arteriolar niche promotes the maintenance and expansion of CSCs within the TME. Direct endothelial DLL4-mediated Notch activation in circulating BCSCs in the arteriolar niche could promote the survival and metastatic potential of cancer cells. On the other hand, TECs with an arteriolar phenotype may directly or indirectly provide signals that actively promote cancer cell stemness for tumor progression. We thus propose that PKD-1 signaling may promote BC stemness by creating an arteriolar niche within the TME. This may be accomplished by stimulating arteriolar differentiation and activating a stemness-related Notch pathway in both TECs and cancer cells. Furthermore, LPA/PKD-1 signaling may mediate ALDH1 expression to contribute to the plasticity of BCSC^32^. Finally, the increased expression of both ALDH1 and CD36 in CSCs may significantly increase metastatic potential (Fig. 8).

We speculate that the arteriolar niche could equip and educate peri-arteriolar and circulating BCSCs by cytokine production and arteriolar blood perfusion to invade surrounding tissues or enter the arterioles. Moreover, this TME-nourished and enhanced CSCs may escape from immune surveillance, survive in the circulating blood, and thus facilitate their spread to the liver and lung. Further investigations are needed to provide deeper insight into whether and how individual factors such as metabolites generated via CD36-mediated fatty acid metabolism or a combination of factors are required to maintain this unique vascular niche and to elucidate specific mechanisms, in which the LPA/PKD-1 signaling axis may be an important player. This study also indicates that inhibition of the LPA/PKD-1 signaling pathway likely disrupts the arteriolar niche for CSCs and eradicate BCSCs. However, this therapeutic effect could be enhanced by combination with activating the CD36 signaling pathway via interaction with its ligand 3TSR (a functional domain of thrombospondin 1) to induce apoptosis^71,72^. Targeting the LPA/PKD-1 signaling pathway may function as a double-edged sword against both vascular and CSC compartments to alleviate therapeutic resistance while also controlling cancer relapse and metastasis. This study also provides mechanistic insight into therapeutic strategies against not only breast cancers but also other types of highly angiogenic cancers such as glioblastoma, lung cancer, malignant melanoma, and pancreatic neuroendocrine cancers.

## Methods

### Study approval

All in vivo experiments were conducted in accordance with the Guide for the Care and Use of Laboratory Animals of the NIH. Animal studies were conducted under approved IACUC from the University of Alabama at Birmingham and the Medical College of Wisconsin. Tumor specimens from human patients with ER^+^ breast cancer were used to perform immunohistochemistry and immunofluorescence experiments without any link to subject-identifiable information.

### Reagents and antibodies

Oleoyl-L-${\rm{\alpha }}$-LPA (L7260) was purchased from Sigma-Aldrich. The PKD inhibitor CRT0066101 (A8679) was purchased from APExBio. The LPA inhibitor Ki16425 (10012659) was purchased from Cayman Chemical Company. Gelatin (9000-70-8) was purchased from Bio-Rad. The reagents for RT-qPCR include the RNeasy Mini Kit (Qiagen), Power SYBR Green PCR Master Mix, and High-Capacity cDNA Reverse Transcription Kit (Applied Biosystems). RT^2^ qPCR Primer Assay and primers for human/mouse CD36, ephrinB2, neuropilin 1, DLL4, PRKD-1 (PKD-1), Oct4, Sox2, and GAPDH (Qiagen and IDT). Antibodies include PKD/PKCμ (D4J1N), Neurophilin1 (D62C7), phospho-Erk (1/2) (D13.14.4E), and human/mouse Erk (1/2) (137F5) (Cell Signaling Technology), ALDH1A1 (AF5869) (R&D Systems) and ephrinB2 (ab131536) and CD36 (ab133625) (Abcam). Antibodies for immunostaining include rabbit anti-human/mouse PKD-1 (4502371) and mouse anti-mouse α-smooth muscle actin(A2547) (Sigma-Aldrich), mouse anti-human/rat CD44 (5640S) and rabbit anti-human/mouse ALDH1A1 (12035S) (Cell Signaling Technology), rabbit anti-human/mouse CD36 (ab133625) (Abcam), AlexaFluor 594 conjugated donkey anti-rabbit IgG (A21207), AlexaFluor 594 conjugated donkey anti-mouse IgG(A21203), AlexaFluor 594 conjugated donkey anti-goat IgG(A11058), AlexaFluor 488 conjugated donkey anti-goat IgG(A11055), AlexaFluor 488 conjugated donkey anti-rabbit IgG(A21206), AlexaFluor 350 conjugated goat anti-mouse IgG (H + L, A11045), and AlexaFluor 488 conjugated goat anti-mouse IgG(A11001) (Invitrogen). VECTASHIELD Antifade Mounting Medium with DAPI (H-1200) was purchased from VECTASHIELD. Transfection reagent TransIT-X2 reagent (MIR6003) was purchased from Mirus. Opti-MEM I reduced-serum medium (51985-034) was from Gibco.

### Cell culture

Human microvascular endothelial cells (HMVECi-D)^45^ were grown in endothelial media containing DMEM medium with 5% MVGS (Gibco, Cat# S00525), and 5% fetal bovine serum (FBS) under 5% CO_2_, at 37 °C. E0771 breast cancer cells were maintained in RPMI 1640 supplemented with 10% FBS and 10 mM HEPES under 5% CO_2_, at 37 °C. MCF7 breast cancer cells were seeded in T75 flasks or 10 cm plates in low glucose Dulbecco’s modified Eagle’s medium (DMEM) containing 10% FBS, 2 mM glutamine, 0.01 mg/ml insulin, and 1% penicillin/streptomycin mix and incubated at 37˚C in an atmosphere of 5% CO_2_. A co-culture of MCF-7 cells transduced with Ds-Red and HMVECi-Ds was plated onto glass-bottom dishes at the ratio of 2:1 for 48 h for cell-to-cell contact analysis.

### Isolation and culture of primary mouse lung endothelial cells

Isolation of lung endothelial cells from control and endothelial-specific pkd-1 knockout mice were modified using a microbeads-based protocol for the isolation of mouse lung endothelial cells^25,41^. Briefly, for each isolation, three to five mice were used for the procedure and the mice were sacrificed by cervical dislocation. The lung tissues were removed and washed in ice-cold PBS, after that the lungs were placed in a cold-serum-free DMEM medium. The lungs were minced and then incubated in 20 mL of Collagenase I (1 mg/mL in DPBS, Sigma C0130) for 45 min in a 37 °C water bath. Every 10 min during this incubation, the tube was gently agitated for a few seconds. After that using a 30 mL syringe attached to a cannula, the suspension was triturated 12 times and then filtering through a 70 µm cell strainer. Cells were spined down, at 400xg for 10 min at 4 °C, the cell pellet was resuspended with 1 mL of cold 0.1% BSA in PBS and incubated with CD31-conjugated dynabeads (Dynabeads Sheep Anti-Rat IgG, Invitrogen 11035) for 10 min on a rotator at room temperature. Subsequently, the beads with cells were separated in a magnetic separator, followed by five washes in cold PBS containing 0.1% BSA. Finally, assumed CD31^+^ endothelial cells were re-suspended and cultured in DMEM media with 5% MVGS (Gibco, Cat# S00525) and 5% FBS and seeded in a gelatin-coated dish.

### Transwell endothelial cell and breast cancer co-culture

Co-culture of endothelial cells with cancer cells without cell-to-cell contact was performed in Transwell cell culture chamber inserts (Corning) with transparent PET membrane of 0.4 µm pores and six-well plates^70^. Briefly, MCF-7 and HMVECi-D were cultured in DMEM (Corning) with 10% FBS and 1% penicillin/streptomycin and EGM-2 Medium (EBM-2 Basal Medium with EGM^TM^-2 MV Microvascular Endothelial Cell Growth Factor, Lonza) respectively for 18 h before starting the co-culture of the two cell lines. MCF-7 cells were seeded in the 6-well plate while the HMVECi-D cells were cultured in the insert at a ratio of 5:1. Totally, 6 × 10^4^ cells were seeded in the insert while 3 × 10^5^ cells were grown in the well. HMVECi-D medium was switched to the MCF-7 culture medium after the two cells were cocultured for 72 h. They were co-cultured in the MCF-7 media for an additional 48 h. Total RNA of MCF-7 cells was then extracted and assayed by RT-qPCR.

### Plasmid transduction and transfection

The lentivirus system was used to transduce genes into E0771 BC cells, a mouse BC stem-like cell (CSC) line derived from E0771 and HMVECs. Five to ten MOI of lentivirus particles containing either luciferase: GFP or Ds-Red or wild-type PKD-1 (PKD-WT) were added to the E0771 or HMVECi-D for the transduction. An EVOS^®^FL cell imaging system or flow cytometry was used to determine transduction efficiency. For plasmid transfection, MCF-7 cells with high PKD-1 expression were incubated in a 6-well plate with a seeding density of 6.0 $\times$ 10^5^ cells/well for 24 h. The scramble control and PKD-1 siRNA (IDT) was then respectively transfected into the cells in Opti-MEM I reduced-serum medium (Gibco) by TransIT-X2 transfection reagent (Mirus) for 6–18 h and replaced with BC culture media containing 10% FBS for an additional 48 h.

### Real-time quantitative-PCR

Gene expression was assessed by real-time quantitative RT-qPCR. The RT2 qPCR primer assays (Qiagen) or qPCR primers (IDT) for the target genes and housekeeping genes were used for PCR reactions. Total RNA was isolated from breast cancer cells or ECs using the RNeasy Mini Kit (Qiagen) and then subjected to RT-qPCR using CFX Connect Real-Time System (Bio-Rad). Genes were assayed with GAPDH or PPIA transcripts that were amplified in separate wells for normalization of variances in input RNA. The relative Ct value was used to compare the fold or quantitative change of mRNA expression.

### Immunoblot assays

Immunoblots of cell lysates were probed with relevant antibodies. Protein concentrations were assayed with a BCA kit (Pierce Chemical) and β-actin was used as a loading control. Cell lysates were separated with commercially ready (Bio-Rad & Fisher) or self-made gel and subjected to Western blots. Densitometry was performed using NIH Image J.

### Immunofluorescence and immunohistochemical assays

Tissues were fixed in 10% formalin for paraffin block preparation, sectioned, and processed, and cells were fixed in 4% PFA for immunohistochemical and immunofluorescence staining with appropriate primary and secondary antibodies, respectively. Immunofluorescence microscopy for cultured cells was performed for the acquisition of images. The detailed information is included in figure legends. Eight human ER^+^ breast cancer samples were used for all immunofluorescence and immunohistochemical analysis.

### Mammosphere formation assays

Mammospheres assays were carried out in six-well plates. A mixture of MammoCult^™^ Proliferation Supplement and MammoCult^™^ Basal Medium was prepared according to the manufacturer’s instructions. Single cells were plated in ultra-low attachment plates (Corning) at a density of 2500 viable cells in 2 ml per well. After 7 days, the colonies were counted under a phase-contrast microscope by two persons. Error bars represent the standard error of the mean of three replicates.

### In vitro extreme limiting dilution and tumorsphere formation assays

MCF-7 cells transfected with scramble control or PKD-1 siRNA were seeded with a decreasing number of cells per well (5000, 1000, 500, 100, 50, 20, 10, and 1 cell) in ultra-low attached 96-well plates. The tumorspheres were cultured in MammoCult™ Medium. The number of wells with/without growth of tumorspheres was quantified after 7 days. The data were analyzed and the log-fraction figure was made using software available at bioinf.wehi.edu.au/software/elda/.

### Breast cancer animal model

Six-week-old female C57BL/6 mice (Jackson Laboratory) were maintained on a chow diet (D12450B, 10 kcal% fat, Research Diets, Inc). Syngeneic breast cancer was grown in the subcutaneous space of mice near the fourth mammary pad by implanting E0771 cells (1 × 10^6^ cells/mouse). Mice-bearing tumors for about three weeks were anesthetized and sacrificed, and tumor tissues were processed for immunohistochemistry and immunofluorescence staining. For LPA treatment experiments, vehicle or LPA (1 mg/kg) was administrated abdominally three days after tumor implantation, with an injection every three days for 18 days. Tumor tissues in the BC model in mice fed with HFD^22^ were also used as controls.

### Statistics and reproducibility

Quantitative data are presented as mean ± SD or SE. Data were analyzed using two-sided unpaired *t* tests with a GraphPad software package. One-way ANOVA was also used to determine whether there are any statistically significant differences between independent groups. A *P* < 0.05 or <0.01 and 0.001 were considered statistically significant or very significant.

### Reporting summary

Further information on research design is available in the Nature Research Reporting Summary linked to this article.

## Supplementary information

Supplementary Information

Description of Supplementary Files

Supplementary Data 1

Reporting Summary

## Data Availability

All data generated or analyzed during this study are included in this published article. Source data is available as Supplementary Data.
