# Supplementary material for: Development of an arteriolar niche and self-renewal of breast cancer stem cells by lysophosphatidic acid/protein kinase D signaling
Source: Commun Biol. 2021 Jun 24;4:780. doi: 10.1038/s42003-021-02308-6 (PMC8225840; doi:10.1038/s42003-021-02308-6)
Supplement: Supplementary file 1 — Supplementary Information [file 42003_2021_2308_MOESM1_ESM.pdf]

Supplementary Figures

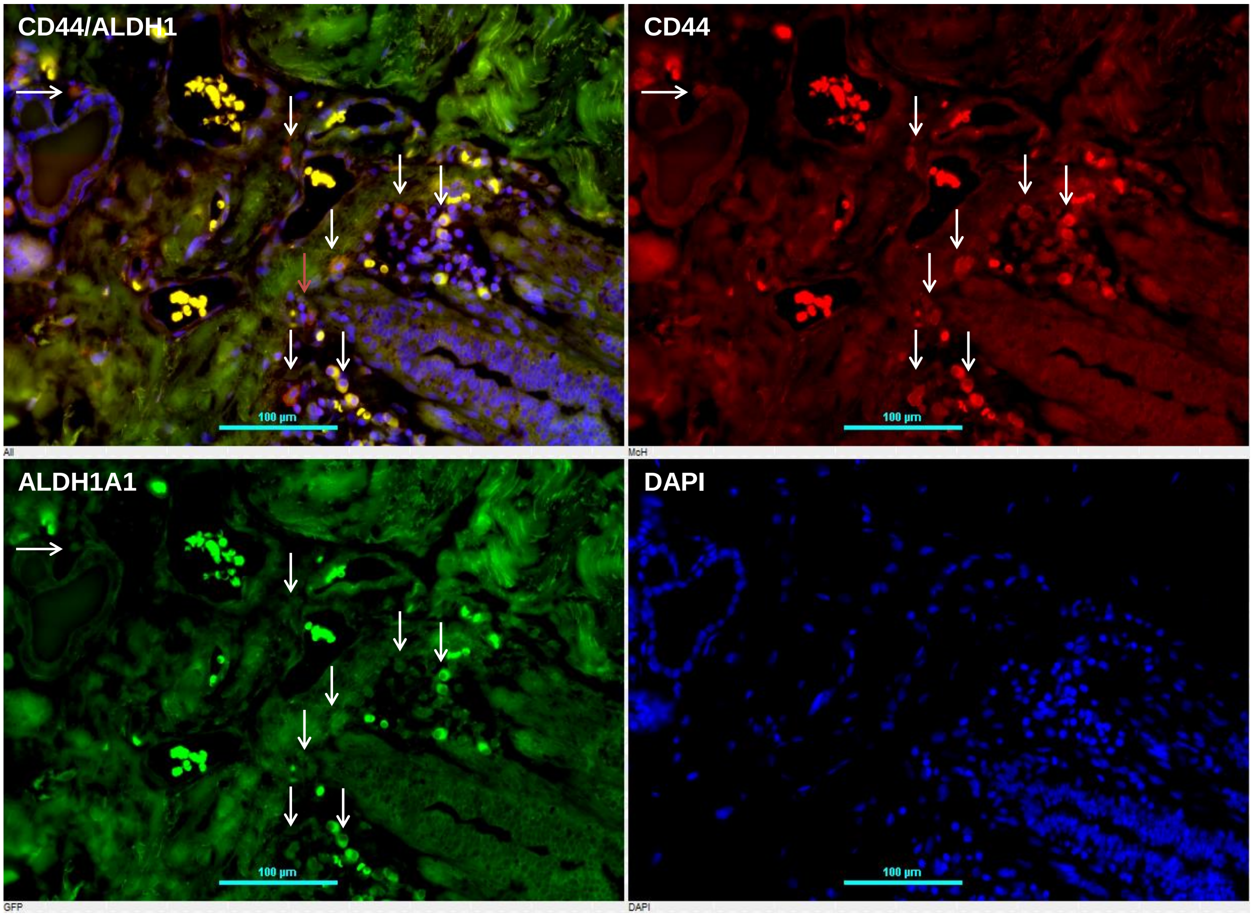

Supplementary Figure 1. Existence of cancer stem-like cells (CSCs) in tissues from patients with ER$^+$ BC. CD44 (red) and ALDH1A1 (green) positive staining in BC cells within the vascular niche (white arrows); co-expression (Yellow); DAPI (blue) for the nuclei; non-specific staining in blood cells (no nuclei). Scale bar = 100 µm.

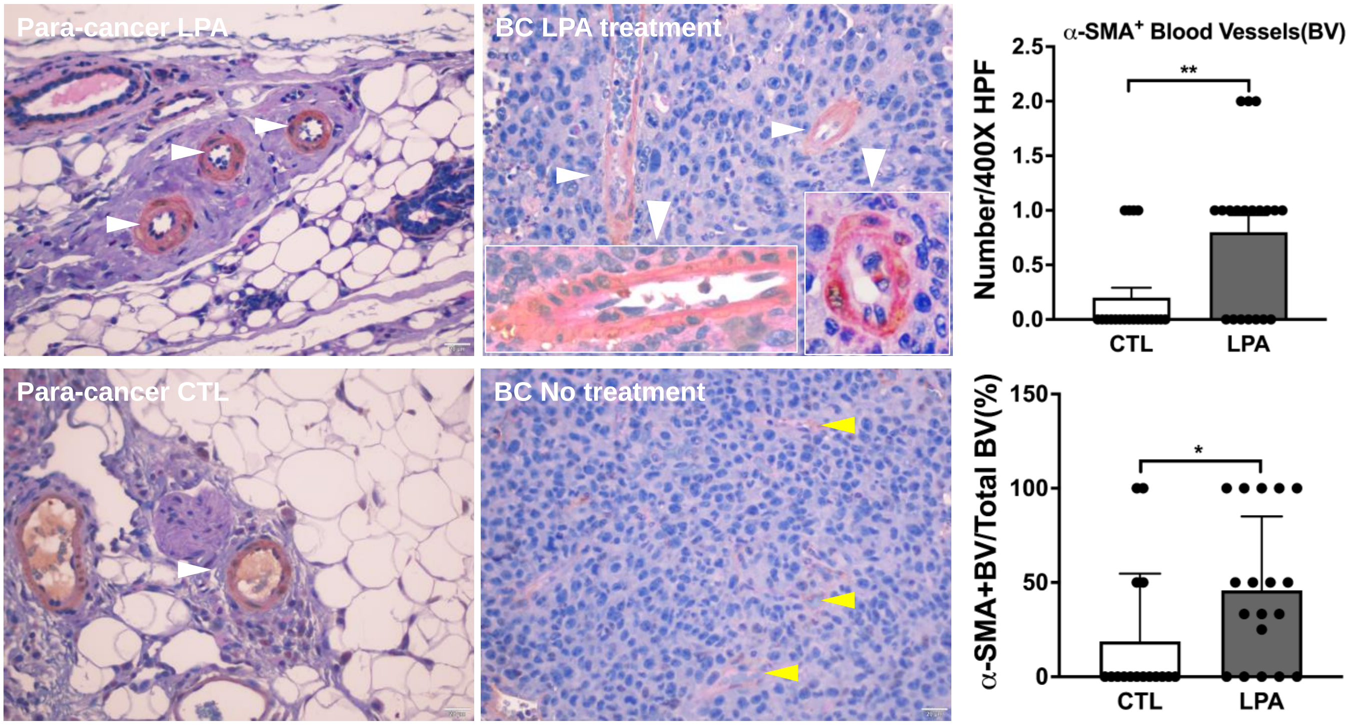

Supplementary Figure 2. LPA treatment increased arteriolar density in BCs. Mice bearing E0771 xenografts treated with LPA (1 mg/kg) showed increased arteriolar density compared to non-treated controls as determined by number of α-SMA blood vessels (BV) and the percent of α-SMA$^+$ BV per total BV. Type of BV was differentiated based on the staining and morphology. Brown and pink staining indicates α-SMA- and basement membrane-positive. White or yellow arrow heads show arterioles or capillaries, respectively. **p*<0.05 or ***p* <0.01 vs control. Scale bar = 10 µm.

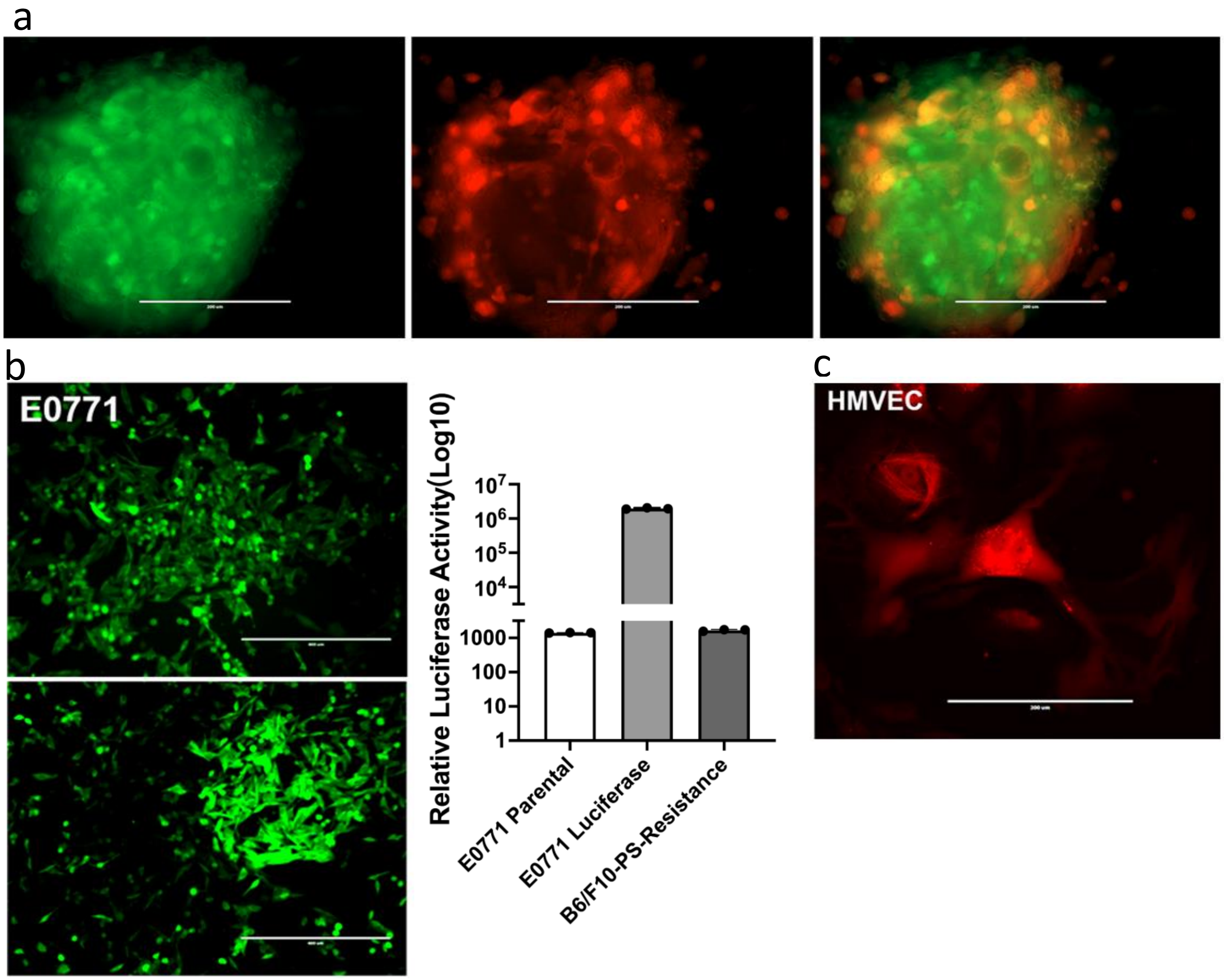

Supplementary Figure 3. Three dimensional co-culture of BC cells and microvascular endothelial cells. **a**. E0771 cells and HMVECs were transduced with GFP or Ds-Red, respectively. Representative images are shown, scale bar = 200 µm. **b.** Representative images of E0771 cells transduced with luciferase and GFP and corresponding luciferase activities showing higher luciferase activity in E0771-transduced cells as compared to parental E0771 cells and melanoma cells (negative control). Scale bar = 400 µm. GFP positive cells were sorted by flow cytometry and luciferase assays were performed using Dual-Luciferase® Reporter Assay System (Promega) with a Wallac 1420 Multilabel Counter from PerkinElmer Life and Analytical Sciences (Turku, Finland). Luciferase activity is shown in the right panel. **c.** An example of HMVECs that were transduced with Ds-Red is shown. Scale bar = 200 µm.

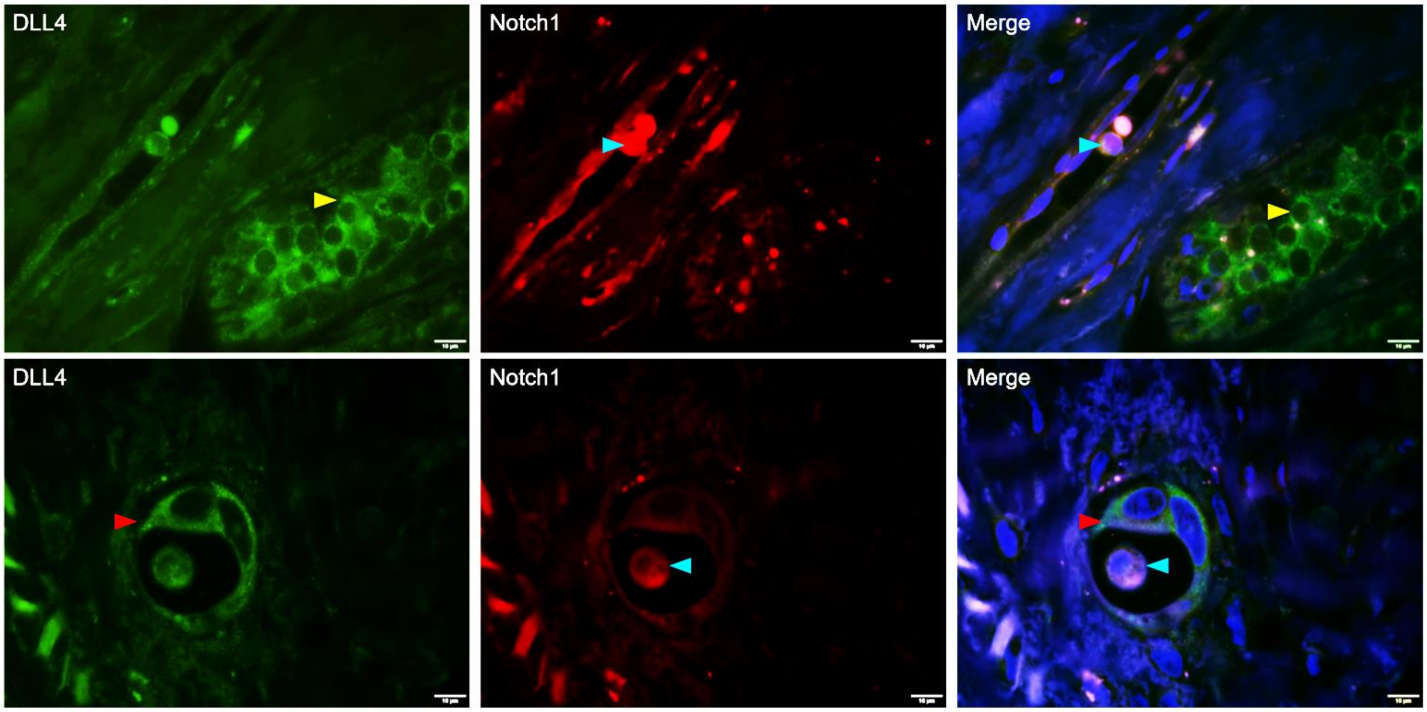

Supplementary Figure 4. DLL-4⁺ and Notch1⁺ BC cells localized to blood vessels in patient tissues. High levels of DLL4 (stained green) were expressed in BC cells located within the cancer nest (yellow arrowheads, upper panel), while BC cells that express higher levels of Notch1 (stained red) and DLL-4 (blue arrowheads) occurred within the capillary (upper panel) or blood vessel composed of DLL-4$^+$ cells (red arrowheads, lower panel). This vascular structure could be vasculogenic mimicry composed of DLL-4$^+$ ECs (red arrows), which are likely derived from DLL-4$^+$ BCSCs as determined by the larger size of the nucleus. Scale bar = 10 µm.

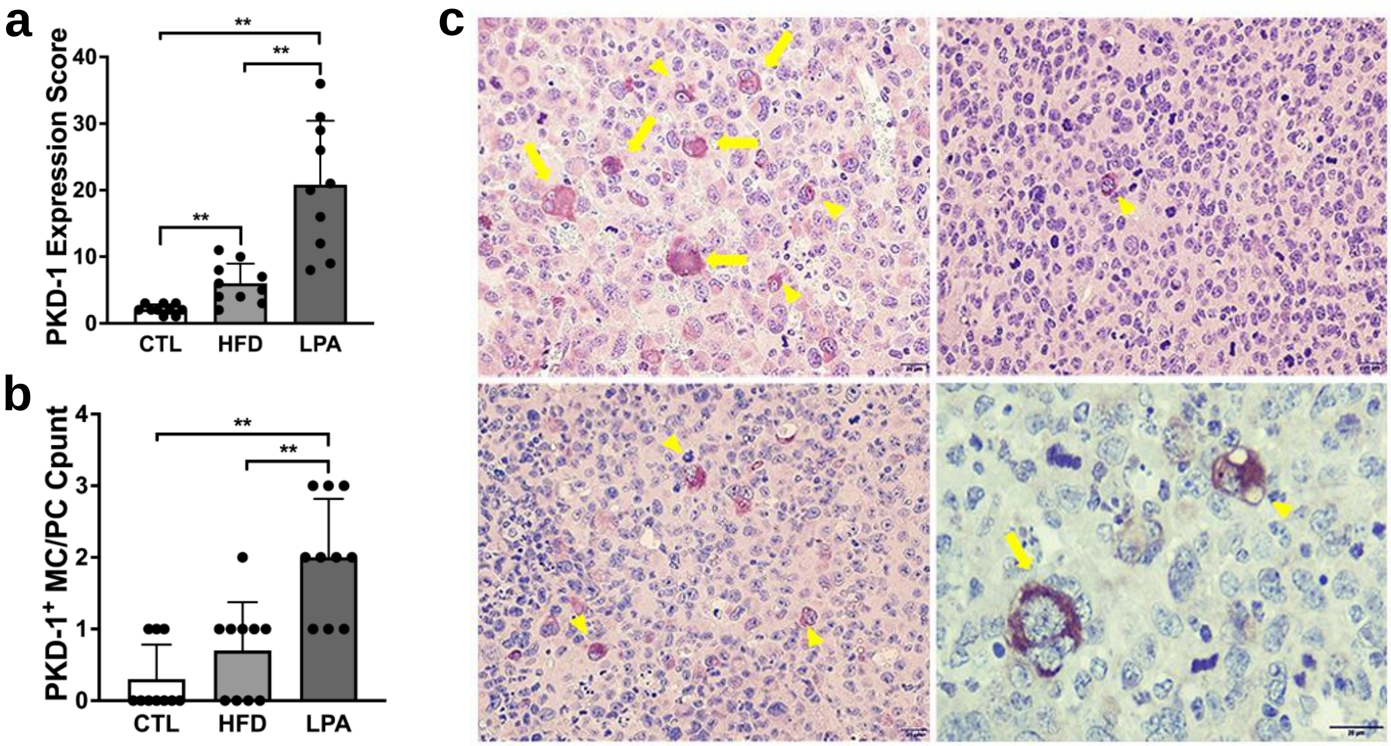

Supplementary Figure 5. LPA treatment increased PKD-1 expression in some BC cells in an ER+ BC mouse model. BL6 mice were subcutaneously implanted with E0771 cells close to the mammary pads and LPA (1 mg/kg) was administered 3 days after implantation for 18 days. **a.** PKD-1 expression score in a subset of BC cells in the control, LPA treatment, and high fat diet (HFD) groups $^{**}p<0.01$; **b.** Higher percentage of PKD-1+ megakaryocyte (MC) and/or polykaryocyte (PC) per high power field (HPF) in response to LPA treatment. Shown are an average score and average number of MC and PC numbers per HPF. High positive (brown-black) cells × 2 + low positive (brown-yellow) cells×1. $^{**}p<0.01$; **c.** Representative Images in LPA-treated E0771 xenografts (top left), diet-induced obesity (top right) and lean control mice (bottom left); and representative magnified image of megakaryocytes and polykaryocyte (bottom right). Arrows and arrow heads denote polykaryocyte and megakaryocyte, respectively. Scale bar = 20 µm.

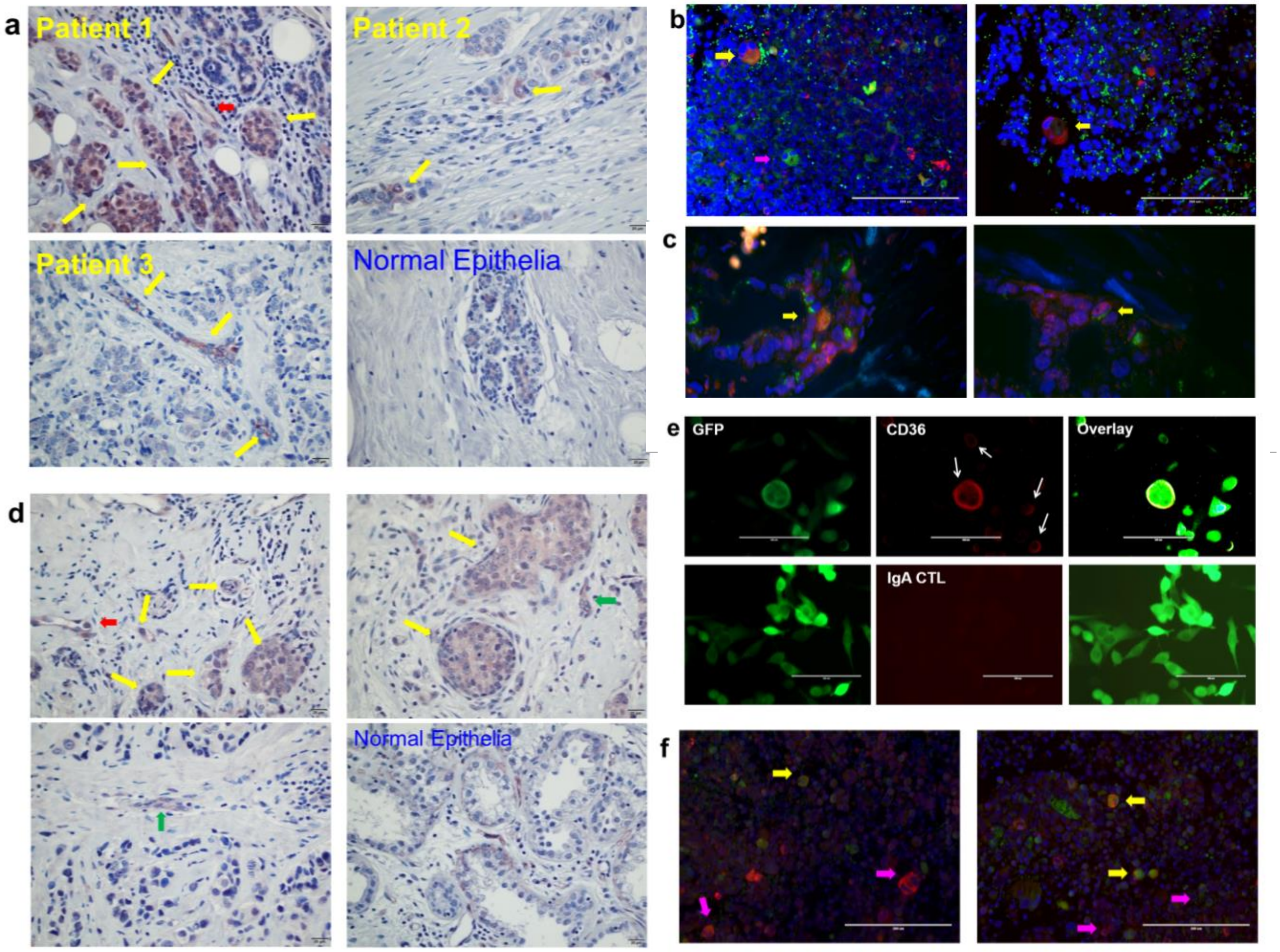

Supplementary Figure 6. A subpopulation of breast cancer stem-like cells positive for PKD-1 and CD36 in tissues from human ER^+ BC patients and a mouse BC model. a. Representative images of the population distribution of clustered or individual PKD-1^+ cells in ER^+ BC tissues, including clusters of BC cells with high levels of PKD-1 expression (upper left panel, yellow arrows), and individual BC cells with moderate PKD-1 expression (upper right and lower left panels). A blood vessel consisting of cells with moderate PKD-1 expression (upper left panel, red arrow). PKD-1 is weakly expressed in normal epithelia (lower right panel). Scale bar = 20 µm. **b.** Representative images that positively express CD44 (green) and PKD-1 (red) in E0771 syngeneic BC tissues; nuclei were stained with DAPI (blue). Yellow arrows indicate $CD44^+$/PKD-1^+ BC cells, and pink arrow shows $CD44^+$ BC cells. PKD-1^+ megakaryocytes or polykaryocytes showed positive CD44 expression. Scale bar = 200 µm. **c.** Tumor tissues from patients with ER^+ BC showed positive expression of CD44 (green) and PKD-1 (red); nuclei stained with DAPI (blue). Bar = 20 µm. **d.** Distribution of a subpopulation of $CD36^+$ cells in human ER^+ BC, including clusters of BC cells with moderate levels of CD36 expression (upper left and right panels, yellow arrows) and individual BC cells with moderate CD36 expression (upper right and lower left panels, green arrows). A blood vessel consisted of cells with moderate CD36 expression (upper left panel, red arrow). CD36 is weakly expressed in normal epithelia (lower right panel). $CD36^+$ BC cells invaded into the stroma and appeared within vascular niche, presenting mesenchymal phenotype with spindle morphology (green arrows). **e.** A few E0771 BC cells transduced with GFP stained for CD36 expression (red). White arrows indicate the $CD36^+$ cells. Representative images are shown, scale bar = 100 µm. **f.** PKD-1 and CD36 were differentially expressed in different E0771 xenograft BC cells with a small subpopulation of cells positively co-expressing both PKD-1 and CD36 (yellow arrows). The cells that expressed either PKD-1 (red) or CD36 (green) were indicated by pink arrows, nuclei stained with DAPI (blue). Representative images are shown, and scale bar = 200 µm.

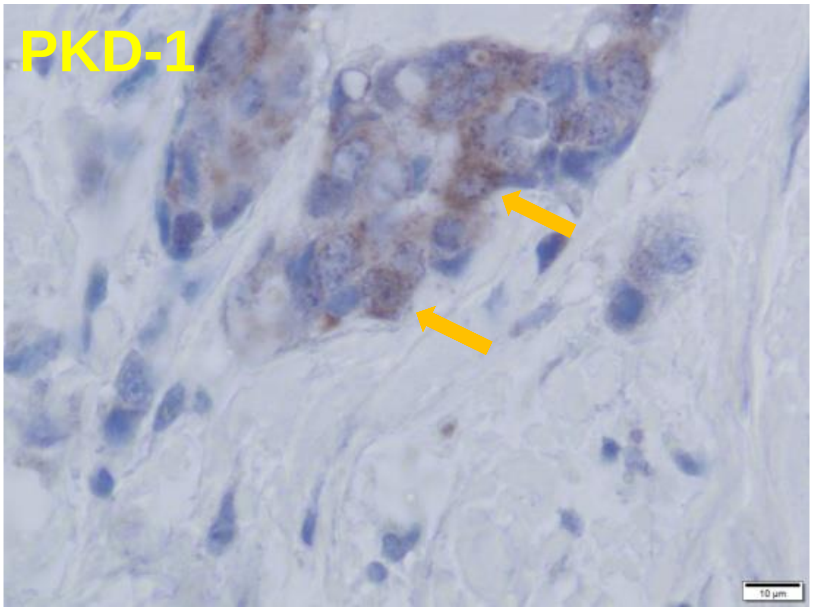

Supplementary Figure 7. PKD-1 was highly expressed in a few BC cells that tend to be at the margin area within the cancer nest (yellow arrows). Scale bar = 10 µm.

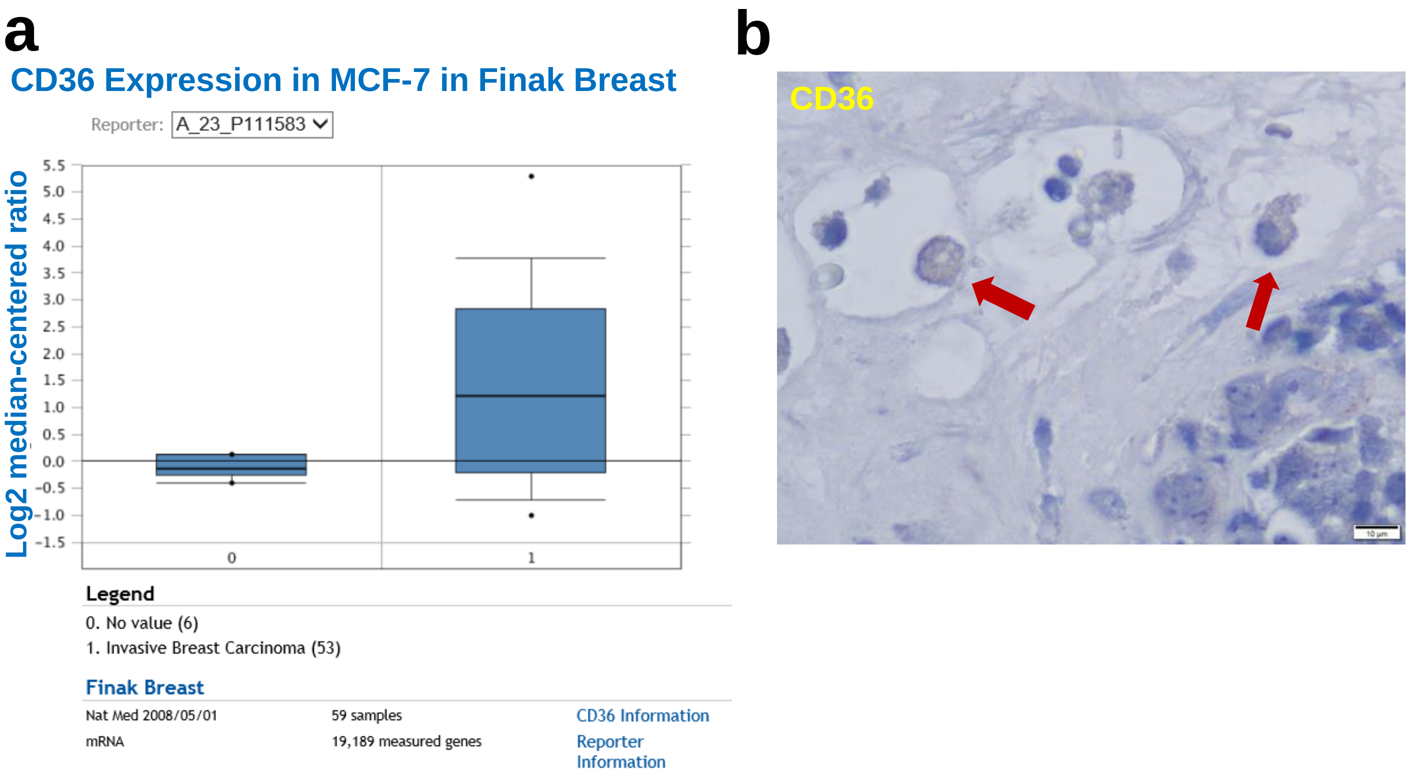

Supplementary Figure 8. CD36 is highly expressed in ER$^+$ breast cancers. a. Oncomine data analysis showed that CD36 expression increased in invasive ER$^+$ BC cancer compared with the control. The data were retrieved from the web link for Oncomine, a Cancer Microarray Database and Integrated Data-Mining Platform: https://www.oncomine.org/resource/login.html. **b**. Representative image of CD36$^+$ expression in ER$^+$ BC tissue. BC with moderate CD36 expression occurred in the lumen of tumor blood vessels (red arrows) in BC tissue from a human patient. Scale bar = 10 µm.
