# Supplementary material for: Development of an arteriolar niche and self-renewal of breast cancer stem cells by lysophosphatidic acid/protein kinase D signaling
Source: Commun Biol. 2021 Jun 24;4:780. doi: 10.1038/s42003-021-02308-6 (PMC8225840; doi:10.1038/s42003-021-02308-6)
Supplement: Supplementary file 2 — Description of Supplementary Files [file 42003_2021_2308_MOESM2_ESM.pdf]

Description of Additional Supplementary Files

File name: Supplementary data 1

Description: Source data for graphs and charts.
