# Supplementary material for: Development of an arteriolar niche and self-renewal of breast cancer stem cells by lysophosphatidic acid/protein kinase D signaling
Source: Commun Biol. 2021 Jun 24;4:780. doi: 10.1038/s42003-021-02308-6 (PMC8225840; doi:10.1038/s42003-021-02308-6)
Supplement: Supplementary file 4 — Reporting Summary [file 42003_2021_2308_MOESM4_ESM.pdf]

nature research

Corresponding author(s): Bin Ren

Last updated by author(s): Mar 10, 2021

Reporting Summary

Nature Research wishes to improve the reproducibility of the work that we publish. This form provides structure for consistency and transparency in reporting. For further information on Nature Research policies, see our Editorial Policies and the Editorial Policy Checklist.

Statistics

For all statistical analyses, confirm that the following items are present in the figure legend, table legend, main text, or Methods section.

n/a	Confirmed	
☐	☒	The exact sample size (*n*) for each experimental group/condition, given as a discrete number and unit of measurement
☐	☒	A statement on whether measurements were taken from distinct samples or whether the same sample was measured repeatedly
☐	☒	The statistical test(s) used AND whether they are one- or two-sided<br>*Only common tests should be described solely by name; describe more complex techniques in the Methods section.*
☐	☒	A description of all covariates tested
☐	☒	A description of any assumptions or corrections, such as tests of normality and adjustment for multiple comparisons
☐	☒	A full description of the statistical parameters including central tendency (e.g. means) or other basic estimates (e.g. regression coefficient) AND variation (e.g. standard deviation) or associated estimates of uncertainty (e.g. confidence intervals)
☐	☒	For null hypothesis testing, the test statistic (e.g. *F*, *t*, *r*) with confidence intervals, effect sizes, degrees of freedom and *P* value noted<br>*Give P values as exact values whenever suitable.*
☒	☐	For Bayesian analysis, information on the choice of priors and Markov chain Monte Carlo settings
☒	☐	For hierarchical and complex designs, identification of the appropriate level for tests and full reporting of outcomes
☒	☐	Estimates of effect sizes (e.g. Cohen's *d*, Pearson's *r*), indicating how they were calculated

Our web collection on statistics for biologists contains articles on many of the points above.

Software and code

Policy information about availability of computer code

Data collection	Microscopy data was collected and analysed with Olympus microscopes and other imaging softwares. qRT-PCR data was collected with CFX Connect Real-Time System (Bio-Rad). Immunoblot data was collected with Bio-Rad Image system and software.
Data analysis	Numerical data was first processed with Microsoft Excel 2010 and after analysed and plotted with Graphpad Prism 8.<br>Microscope images were processed and analysed/quantified with Image J v1.52a. Adobe Photoshop CC 20.0.4 were used for downstream image analysis and illustration.

For manuscripts utilizing custom algorithms or software that are central to the research but not yet described in published literature, software must be made available to editors and reviewers. We strongly encourage code deposition in a community repository (e.g. GitHub). See the Nature Research guidelines for submitting code & software for further information.

Data

Policy information about availability of data

All manuscripts must include a data availability statement. This statement should provide the following information, where applicable:

- Accession codes, unique identifiers, or web links for publicly available datasets
- A list of figures that have associated raw data
- A description of any restrictions on data availability

The source data and statistical information underlying all figures and charts is provided in the source data file and supplementary data file. All other data supporting the study findings are available from the corresponding author upon request. This includes additional raw data such as unprocessed original pictures and independent replicates, which are not displayed in the manuscript but are included in the data analysis in the form of graphs.

Field-specific reporting

Please select the one below that is the best fit for your research. If you are not sure, read the appropriate sections before making your selection.

- [x] Life sciences
- [ ] Behavioural & social sciences
- [ ] Ecological, evolutionary & environmental sciences

For a reference copy of the document with all sections, see nature.com/documents/nr-reporting-summary-flat.pdf

Life sciences study design

All studies must disclose on these points even when the disclosure is negative.

Sample size	Sample size was determined taking into account the expected experimental variability or a posteriori based on the statistical analysis of the data. Briefly, two groups of samples with a Gaussian distribution were compared by unpaired two-tailed Student T-test. Comparisons among more than two groups were made by ANOVA test. Graphs represent mean +/- SD as indicated, and differences were considered significant at $p < 0.05$ as indicated. All calculations were done in Excel and final datapoints analysed and represented with GraphPad Prism.
Data exclusions	Data was excluded only if technical problems were detected. These include technical problems detected after immunostaining or unexpected animal death.
Replication	We repeated most experiments at least twice to guarantee maximum reproducibility. In addition, the main conclusions are supported by several different experiments.
Randomization	Animals were treated randomly before tissues collection and analysis. Animals/tissues were selected for a posteriori analysis based on their genotype. For in vitro experiments samples were allocated randomly in each experiment.
Blinding	In the majority of cases, investigators were not blinded during data collection or analysis. Image software was used to analyse the microscopy data in an automatic and objective manner. All experiments in the paper were quantified utilizing standardized experimental controls and quantitative methods to avoid bias.

Reporting for specific materials, systems and methods

We require information from authors about some types of materials, experimental systems and methods used in many studies. Here, indicate whether each material, system or method listed is relevant to your study. If you are not sure if a list item applies to your research, read the appropriate section before selecting a response.

Materials & experimental systems

n/a	Involved in the study	
[ ]	[x]	Antibodies
[ ]	[x]	Eukaryotic cell lines
[x]	[ ]	Palaeontology and archaeology
[ ]	[x]	Animals and other organisms
[x]	[ ]	Human research participants
[x]	[ ]	Clinical data
[x]	[ ]	Dual use research of concern

Methods

n/a	Involved in the study	
[x]	[ ]	ChIP-seq
[x]	[ ]	Flow cytometry
[x]	[ ]	MRI-based neuroimaging

Antibodies

Antibodies used	All detailed information is contained in MATERIALS AND METHODS section
Validation	All antibodies used are commercially available and have been pre-validated by the companies and us. They all gave immunostaining or immunoblotting results according to what was expected from their previously published tissue expression pattern or the predicted immunoblot target protein size.

Eukaryotic cell lines

Policy information about cell lines

Cell line source(s)	HMVEC-D was derived from dermal HMVEC originally from Lonza.
Authentication	Cells lines were not formally authenticated, but they had the reported phenotype and biology.
Mycoplasma contamination	Cells were routinely checked for mycoplasma contamination and were found to be negative.

Commonly misidentified lines (See ICLAC register)	No commonly misidentified cell lines were used.

Animals and other organisms

Policy information about studies involving animals; ARRIVE guidelines recommended for reporting animal research

Laboratory animals	Jackson Laboratories. Approved by MCW and UAB IACUC.
Wild animals	No wild animals were used in the study.
Field-collected samples	No field collected samples were used in the study.
Ethics oversight	All in vivo experiments were conducted in accordance with the Guide for the Care and Use of Laboratory Animals of the NIH. Animal studies were conducted under approved IACUC from University of Alabama at Birmingham and Medical College of Wisconsin. Tumor specimens from human patients with ER+ breast cancer were used to perform immunohistochemistry and immunofluorescence experiments without any link to subject identifiable information.

Note that full information on the approval of the study protocol must also be provided in the manuscript.
